# Convenient Genetic Encoding of Phenylalanine Derivatives through Their α-Keto Acid Precursors

**DOI:** 10.3390/biom11091358

**Published:** 2021-09-14

**Authors:** Li Liu, Bohao Wang, Sheng Li, Fengyuan Xu, Qi He, Chun Pan, Xiangdong Gao, Wenbing Yao, Xiaoda Song

**Affiliations:** 1 Jiangsu Key Laboratory of Druggability of Biopharmaceuticals, Department of Biochemistry, School of Life Science and Technology, China Pharmaceutical University, Nanjing 210009, China; pluto_aars@163.com (L.L.); bhwang1994@foxmail.com (B.W.); 17786650010@163.com (S.L.); becomethe@163.com (F.X.); hqawjnanjing@163.com (Q.H.); 2 Jiangsu Provincial Key Laboratory of Critical Care Medicine, Department of Critical Care Medicine, School of Medicine, Southeast University, Nanjing 210009, China; panchun1982@gmail.com

**Keywords:** keto acids, biotransform, evolution

## Abstract

The activity and function of proteins can be improved by incorporation of non-canonical amino acids (ncAAs). To avoid the tedious synthesis of a large number of chiral phenylalanine derivatives, we synthesized the corresponding phenylpyruvic acid precursors. *Escherichia coli* strain DH10B and strain C321.ΔA.expΔPBAD were selected as hosts for phenylpyruvic acid bioconversion and genetic code expansion using the *Mm*PylRS/^pyl^tRNA_CUA_ system. The concentrations of keto acids, PLP and amino donors were optimized in the process. Eight keto acids that can be biotransformed and their coupled genetic code expansions were identified. Finally, the genetic encoded ncAAs were tested for incorporation into fluorescent proteins with keto acids.

## 1. Introduction

Natural proteins are typically produced from twenty different amino acids as buildingNatural proteins are typically produced from twenty different amino acids as building blocks, sometimes in combination with pyrrolysine and selenocysteine. To expand the structural and functional diversity of proteins, non-canonical amino acids (ncAAs) have been introduced into proteins by reprogrammed protein translation [1,2,3,4,5,6]. In the past two decades, this technology, known as genetic code expansion (GCE), was further developed [7,8]. This powerful technology can incorporate ncAAs into proteins in a site-specific manner. By introducing both the orthogonal aminoacyl-tRNA synthetase (aaRS) and the nonsense suppressor tRNA into a cell, a ncAA is loaded onto the suppressor tRNA, whose anti-codon recognizes the amber nonsense codon [9] or even a quadruplet codon [10]. The cell’s translation machinery then introduces the ncAAs into the stop codon or quadruplet codon of the target gene.

In conventional GCE, the desired ncAAs are synthesized in their proper chiral form and then added to the culture medium from which they are taken up by the cells. It can be difficult and time consuming to synthesize a large number of L-ncAAs with a chiral center in the α-carbon; this step is described as the Achilles’ heel in synthetic biology [11]. In addition, uptake of the ncAAs can be limited by the available native amino acid transporters [12]. Alternatively, the ncAAs are synthesized by the cells in situ, from inexpensive precursors added to the culture medium. This overcomes complicated feeding schemes and the production of costly substrates, but it can also be a challenge [13].

The coupling of simple metabolic engineering with reprogrammed translation has been demonstrated by several research groups [14]. In 2003, Mehl et al. were the first to report the generation of *p*-aminophenylalanine in situ via a chloramphenicol biosynthetic pathway [15]. Later, Ou et al. [16] and Ehrlich et al. [17] transplanted a partial pyrrolysine biosynthetic gene into *E. coli* to produce genetically encoded novel pyrrolysine analogues with functional groups. In 2014, four ncAAs were synthesized from their corresponding α-keto acids by introducing glutamine:phenylpyruvate aminotransferase (GlnAT) from *Thermus thermophilus* HB8 into *Escherichia coli* BL21, which enabled their incorporation into proteins [18]. Kim et al. genetically incorporated L-dihydroxyphenylalanine (DOPA) that was biosynthesized by a tyrosine phenol lyase [19]. In 2016, Exner et al. described in situ production of S-allyl cysteine from allylthiol that was incorporated into proteins by pyrrolysyl-tRNA synthetase [20]. The group of Budisa reported the biosynthesis of S-allyl-L-homocysteine from allyl mercaptan, coupled with the incorporation of ncAAs into proteins by auxotrophic cell lines [21]. Work by Di Salvo and collaborators showed how the hijacking of methionine biosynthesis of *E. coli*, via the introduction of acetylated homoserine (two genes from a *Corynebacterium* were actually used for direct sulfhydration), can be used for in situ production of different ncAAs [22,23,24]. Phosphothreonine, which has an impenetrable cell membrane, could be biosynthesized and genetically encoded into protein [25], and, recently, it was shown that the cysteine biosynthetic enzymes could be hijacked to introduce multiple homophenylalanine derivatives [26].

**Figure 1:**
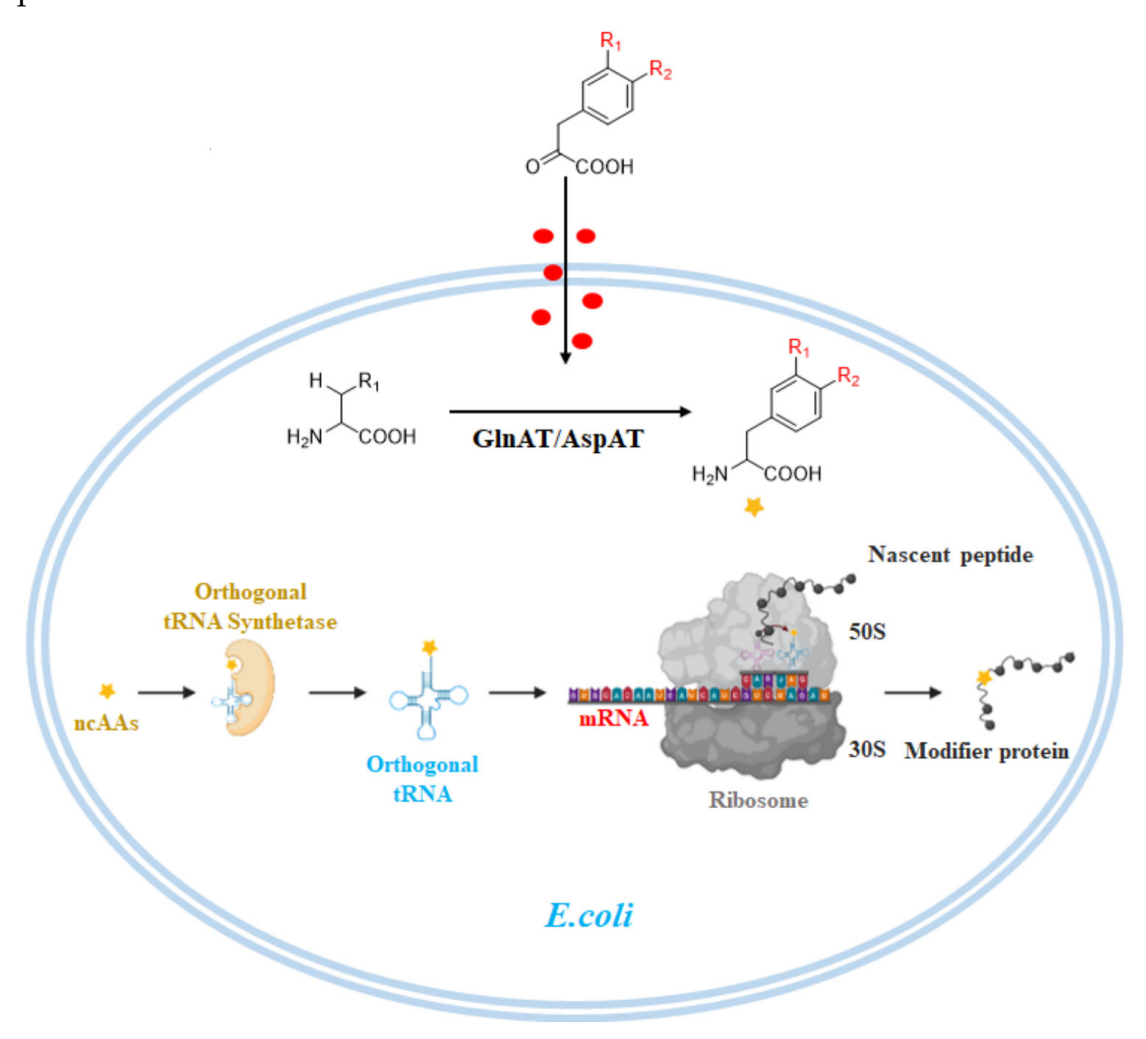
Simple intracellular metabolic conversion (biotransformation) of phenylalanine derivatives by their α-keto acid precursors. Incorporation of ncAAs from phenylpyruvate derivatives into proteins is enabled by the orthogonal aminoacyl-tRNA synthetase, which aminoacylates an orthogonal tRNA with the ncAA. The aminoacylated orthogonal tRNA recognizes the amber codon in the mRNA of a protein and introduces the ncAA at this position.

We intend to develop a strategy to introduce an L-Phe derivative into protein (Figure 1). For this, we screened host strains that could biotransform keto acids into ncAAs without a need to introduce aminotransferases. A series of keto acids were synthesized that could serve as precursors for L-Phe derivatives, and those keto acids that could be recognized by biotransformed coupling ncAAs were identified. Their incorporation into green fluorescent protein (GFP) was used as a model to test fluorescence enhancement following the introduction of ncAAs. The described strategy can be generally applied to enable synthetic protein evolution.

## 2. Materials and Methods

### 2.1. Chemicals

Non-canonical amino acid L-4-methoxy-phenylalanine (*p*-MeO-F) was purchased from Aladdin (Shanghai, China, # M117085). All used aldehydes and hydantoin were of analytical grade and purchased fromEnergy-chemical (Shanghai, China). All other used chemicals were of analytical grade.

### 2.2. Synthesis of Keto-Acids

**Scheme 1:**
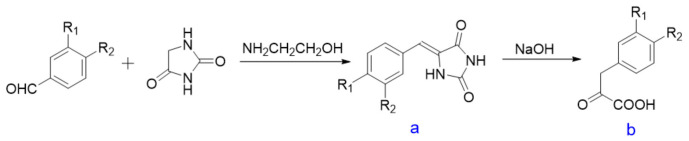
The synthesis pathway for production of α-keto acid derivatives.

All keto acid derivatives were synthesized according to a general reaction scheme summarized in Scheme 1. The derivative *p*-methoxyphenylpyruvate (*p*-MeO-PPA) was used as a proof of principle to introduce the synthesis process of keto acids and to optimize the process. The keto acids were produced by combining 20 g hydantoin (0.2 mol), 60 mL of double-distilled water, and 12 mL ethanolamine into a round bottom flask (in this order). The mixture was stirred and heated to 90 °C until the hydantoin was completely dissolved. Following the addition of 27.23 g (0.2 mol) *p*-methoxybenzaldehyde, stirring was continued at the same temperature for 5 h. After cooling, the precipitate was filtered and placed in a vacuum drying oven for 48 h to obtain the crude product (Z)-5-(4-methoxybenzylidene)imidazolidine-2,4-dione. Recrystallisation was performed with ethanol, which resulted in a white powder. After characterization, 20 g of the product was combined with 100 mL 4 M NaOH in a round bottom flask and heated to reflux at 90 °C for 3 h. After cooling, the pH was adjusted to 8.0 with 1 mol/L hydrochloric acid for precipitation. The precipitate was dried and used directly without purification. The purity was about 80–90%, and since keto acids are unstable in air, it was kept under N_2_ at −20 °C. Synthesis of the other derivatives used in this study is described in Figures S1–S26.

### 2.3. Bacterial Strains, Plasmids

Strains *E. coli* DH10B, BL21AI and Top10F were purchased from Weidi (Shanghai, China, #DL1070, # EC1004, # DL1011). *E. coli* strain C321.ΔA.exp was a gift from Prof. George Church (Addgene # 49018) [27]. The plasmids pKD3, pKD46 and pCP20 were produced in house. The plasmids pBAD-eGFP, pBAD-eGFP(149TAG) and pEvolve-MmRS were constructed in our laboratory. The latter ensured use of the amber codon for introduction of ncAAs in position 149 of GFP.

### 2.4. Construction of E. coli C321.ΔA.expΔP_BAD_ Defective in Arabinose Metabolism

A standard lambda red recombination system [28] was used for knocking out the *araBAD* promoter (P_BAD_) located at genome position 69897–70191 of *E. coli* C321.ΔA.exp (genome sequence accession number CP006698.1) [29]. The linear fragment contained flippase recognition target (FRT) sites. The chloramphenicol resistance gene *cmR* was amplified from plasmid pKD3 with the primers 321-KO-F and 321-KO-R (5′-GGGATCATTTTGCGCTTCAGCCATACTTTTCATACTCCCACCATTCAGAGATCCTCCTTAGTTCCTATTCC, 5′-CTCCATCCAAAAAAACGGGTATGGAGAAACAGTGAGAGTTGCGATAAAAGTGTAGGCTGGAGCTGCTTC).

The purified PCR product was electroporated into electrocompetent C321.ΔA.exp cells that contained the pKD46 plasmid. After induction by 0.2% arabinose, the bacteria were resuspended and spread on LB plates containing 25 μg/mL chloramphenicol (Cm). Transformants were selected and verified with colony PCR. Positive strains were transformed with pCP20 plasmid and cultured for 15 h at 42 °C to cure the *cmR* plasmid. The final mutant C321.ΔA.expΔP_BAD_ was verified by colony PCR followed by sequencing. The efficiency of protein expression was tested with plasmid pBAD-eGFP.

### 2.5. Fluoresce Tests for eGFP Containing ncAAs

The plasmids pEvolve-MmRS and pBAD-eGFP(149TAG) were co-transformed into *E. coli* strain DH10B. A single colony was collected and cultured overnight in 20 mL LB medium (220 rpm) at 37 °C with Amp/Cm selection. The cultured bacteria were diluted 1:100 with LB medium and cultured at 37 °C at 220 rpm for 3 h to an OD_600_ of 0.6. Then, 0.15% arabinose was added and the bacterial suspension was divided into aliquots of 3 mL pro tube. To each tube, either 1 mM ncAA or 5 mM keto-acid (final concentrations) was added and culturing was continued at 37 °C (220 rpm) for 10 h. Samples (800 μL) were then collected for fluorescence detection. After centrifugation at 12,000 rpm for 2 min, the supernatant was discarded, and 200 µL BugBuster protein extraction reagent (Merck Millipore) was added. After mixing, the mixture was left to stand at room temperature for 20 min and then 600 µL PBS solution was added; 200 µL of the solution was used for detection of fluorescence intensity with a multifunctional microplate reader. The excitation wavelength was 480 nm and the emission wavelength was 530 nm. The remaining bacterial solution (200 µL) was used to measure OD_600_. All experiments were performed in triplicate.

### 2.6. Optimization of GFP Production

*E. coli* DH10B harboring the Mm-RS/tRNA system, cultured in the presence of *p*-MeO-PPA, was used to optimize eGFP fluorescence. The concentration of *p*-MeO-PPA in the medium was varied (0.01, 0.05, 0.1, 0.5, 1.0, 5.0, and 10 mM). The addition of Glu and of Asp at concentrations of 0.5, 1.0, 2.0, 3.0, and 5.0 mM, as well as the addition of pyridoxal 5′-phosphate (PLP) at 10, 50, and 100 µM, was also tested.

### 2.7. eGFP Purification for MS Detection

Bacterial cultures obtained with ncAAs or keto acids as described under 2.5 were centrifuged at 8000 rpm for 10 min. To the bacterial pellet, 50 mM Tris-HCl (pH 8.0) was added to give approximately 1g bacteria in 10 mL. The solution was subjected to high-pressure homogenization at 1500 bar and 4 °C (repeated four times). Following centrifugation at 12,000 rpm for 20 min the supernatant was passed through a 0.45 um filter. Q anion columns were used for a first round purification. The supernatant containing eGFP was loaded at a rate of 2.0 mL/min and equilibrated with Tris-HCl (pH 8.0). Then, NaCl at concentrations of 50, 100, 200, 300, 400, and 500 mM was used to elute the protein. The eGFP solution was collected and characterized by 15% SDS-PAGE before the round of Ni affinity purification. For this, the eGFP solution was loaded at a rate of 2.0 mL/min onto the Ni column (General Electric). After equilibration with 500 mM NaCl Tris-HCl (pH 8.0), imidazole at different concentrations (20, 50, 100, 200, 300, 400, and 500 mM) was used to elute eGFP. The eluted eGFP was analyzed by 15% SDS-PAGE and Western blots using commercial anti-GFP antibodies (Proteintech). The purified eGFP protein solution was concentrated with a 10 kD ultrafiltration tube and centrifuged at 4000× *g* at 4 °C and dialyzed to H_2_O for MS detection.

## 3. Results

### 3.1. Construction of Escherichia coli Strain C321.ΔA.expΔPBAD

The *E. coli* C321.ΔA.expΔPBAD-Cm PBAD knockout strain was constructed as described in the Materials and Methods and selected on Cm plates. Obtained colonies were verified by PCR. After the target fragment was integrated into the genome, the expected 1500 bp amplicon was observed in lanes 2, 6, and 7 (Figure S27). Lanes 1 and 4 seemed to comprise mixed bacteria, while lanes 3 and 5 contained knockout failure strains. The bacteria of lane 2 were selected for PCP20-induced *cmR* curation. As shown in Figure S28, similar to the positive control in lane), all cured bacteria produced the shorter 847 bp fragments, and elimination of the Cm resistance gene was also confirmed by the culture on agar plates. To verify BAD expression in the PBAD knockout host strain, the plasmid pBAD-eGFP containing the *P_BAD_* promoter was transformed into C321.ΔA.exp and into C321.ΔA.expΔP_BAD_. After induction with arabinose, the fluorescence upon ultraviolet excitation was stronger for the resulting transformants of C321.ΔA.expΔP_BAD_ compared to those obtained with C321.ΔA.exp (Figure S29).

### 3.2. Screening for Bacterial Strains Utilizing the Keto-Acids

**Figure 2:**
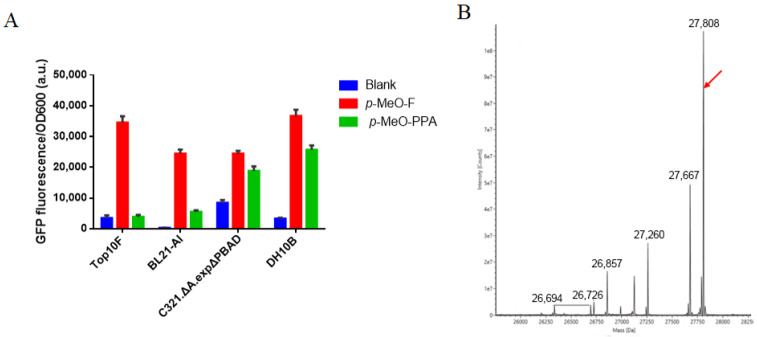
Comparison of the eGFP fluorescence of different *E. coli* strains supplemented with *p*-MeO-F or *p*-MeO-PPA. (**A**). The measured total eGFP fluorescence intensity is the result of the biotransformation of the supplemented keto acids. In vivo amber suppression activity is enabled in strains contain plasmids pBAD-eGFP(149TAG) and pEvolve-MmRS/tRNA_CUA_, using absence of *p*-MeO-PPA. (**B**). Electrospray ionization mass spectrometry of purified eGFP obtained from *E. coli* cultured in presence of *p*-MeO-PPA confirmed the incorporation of *p*-MeO-F. The error bars show the mean and SEM of three independent replicates.

The aim of this study was to enable keto acid biotransformation by genetic coupling of ncAAs encoded with orthogonal Mm-TyrRS. To determine the most suitable host, the following *E. coli* strains with an arabinose metabolic defect were compared: strains DH10B, Top10F, BL21AI, and the constructed C321.ΔA.expΔP_BAD_. These were cultured in the presence of *p*-MeO-PPA or *p*-MeO-F (as a positive control). Without *p*-MeO-F or *p*-MeO-PPA, little fluorescence was detected in all bacteria, with a background of detection for the strain C321.ΔA.expΔP_BAD_. All strains could introduce *p*-MeO-F into eGFP, as indicated by the strong fluorescence signal when *p*-MeO-F was added to the culture medium (Figure 2A). However, following the addition of *p*-MeO-PPA, no fluorescence was found in the strains Top10F and BL21AI, as these strains could not perform keto acid biotransformation. In contrast, the eGFP fluorescence in the strains DH10B and C321.ΔA.expΔPBAD was significantly increased with *p*-MeO-PPA as compared to the control (Figure 2A). These data suggest that the strains DH10B and C321.ΔA.expΔPBAD can efficiently transform *p*-MeO-PPA into *p*-MeO-F. The strongest fluorescence was obtained with *E. coli* DH10B cells, so this strain was chosen as the most suitable host for single-site ncAA introduction. The strain C321.ΔA.expΔP_BAD_ would be more suitable to test the introduction of multiple ncAAs. To confirm that *p*-MeO-PPA had indeed been converted to *p*-MeO-F and had been introduced into eGFP, the eGFP expressed by *E. coli* DH10B in the presence of *p*-MeO-PPA was purified. The SDS-PAGE results of the purification procedure are summarized in Figure S30. Electrospray ionization mass spectrometry was used to confirm the molecular weight of the protein. The theoretical molecular weight of eGFP containing the ncAA is 27809.32 Da, and the actual molecular weight was determined as 27,808 Da (Figure 2B). This confirmed that a single *p*-MeO-F amino acid had been introduced. The mutation Tyr149TAG resulted in its introduction at position 149 of the eGFP protein.

### 3.3. Effects of Substrate Concentration, Cofactor PLP and Amino Donor

**Figure 3:**
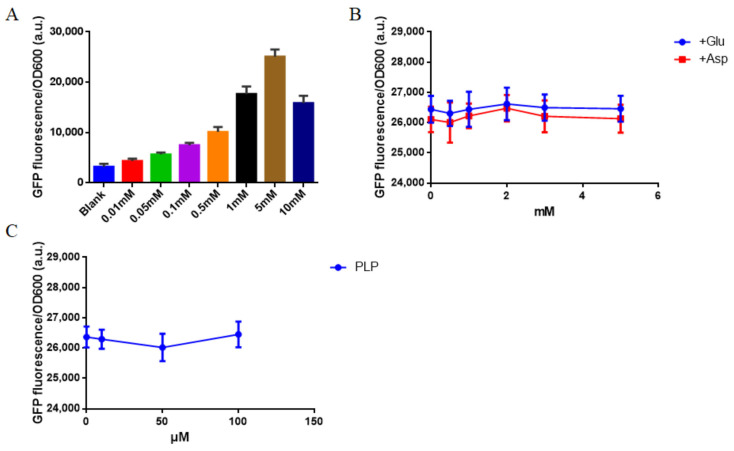
Optimizing the conditions for incorporation of the ncAA into eGFP using *E. coli* DH10B. Various concentrations of *p*-MeO-PPA were added to the medium (**A**), giving the highest expression efficiency at 5 mM. Addition of Glu or Asp as amino donor (**B**) or addition of PLP (**C**) had no effect on the total EGFP fluorescence intensity. The data are presented as mean ± SD of triplicate experiments.

It was determined whether the keto acid biotransformation coupled with genetic ncAA encoding could be optimized. First, the substrate concentration was varied, and the effect on fluorescence generation was determined. This was carried out using *E. coli* DH10B harboring the Mm-RS/tRNA system, to which *p*-MeO-PPA was added to the culture medium at increasing concentrations, from 0.01 to 10 mM. The average fluorescence value increased with increasing concentrations to reach a maximum of 5 mM (Figure 3A). At a concentration of 10 mM the keto acid increased the acidity of the medium too much, and bacterial growth was inhibited, as indicated by lower OD_600_ values (results not shown). We chose 5 mM as the final concentration for the subsequent biotransformation of other keto acids.

Generally, the transamination of phenylpyruvate requires the participation of pyridoxal 5′-phosphate (PLP) as a cofactor, and an amino donor, such as Glu or Asp, can also be required [30]. Thus, PLP and Glu or Asp are often added to large-scale biotransformation of keto acids [31,32]. The effect of adding these components separately to the culture medium was determined. At the tested concentrations between 0.5 and 5 mM, the addition of neither Glu nor Asp had an obvious effect on the fluorescence of eGFP (Figure 3B). Similarly, the addition of 10, 50, or 100 µM PLP did not increase the fluorescence of eGFP (Figure 3C). Accordingly, further screening of fluorescence was performed in the presence of 5 mM keto acids, but no other additions were found.

### 3.4. Screening for Synthesised Keto Acids Introduction

**Figure 4:**
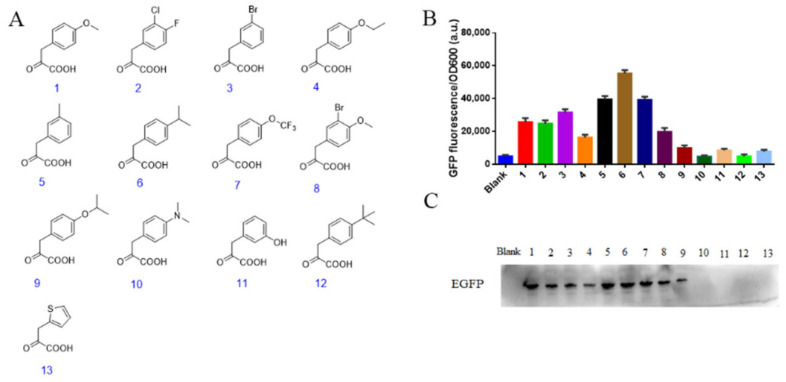
Incorporation of different keto acid derivatives into GFP by *E. coli* DH10B containing the *Mm*PylRS/^pyl^tRNA_CUA_ pair. (**A**) Thirteen different keto acid derivatives were synthesized. (**B**) Their incorporation into eGFP by *E. coli* DH10B varied, and eight resulted in high enzymic activity. (**C**) Production of eGFP was confirmed by Western blot analysis for nine keto acids.

To establish which keto acids can be used by *E. coli* DH10B, 13 different keto acids were synthesized (Figure 4A), and these were tested using the GFP reporter assay. Since the amino acid at position 149 is located at a distance from the core of the chromophore, a change in amino acid at this position would be unlikely to change the fluorescence activity. Therefore, the variation in the obtained fluorescence was taken as a measure of how efficiently the keto acids could be recognized by the transaminase and aminoacyl tRNA synthetase. Figure 4B shows that nine keto acids (derivatives from #1 to #9 of Figure 4A) could be recognized. Expression of eGFP was confirmed by Western blots of cell lysates (Figure 4C). *p*-Isopropyl phenylpyruvate (#6) resulted in the highest fluorescence value of 60,000. Numbers 1, 2, 3, 5 and 7 displayed an average of 20,000 to 40,000 fluorescence values. The large side chain of tert-butyl in #12 was not recognized and eGFP was not expressed. Although #9, containing an isopropyloxy group, was incorporated (lane 9, Figure 4C), fluorescence of the produced eGFP was weak (Figure 4B). The para-dimethylamino group in #10 and the meta-hydroxy group in #11 were strongly hydrophilic, which may have hampered their recognition. The keto acid with thiophene (#13) was also not introduced into the protein.

**Figure 5:**
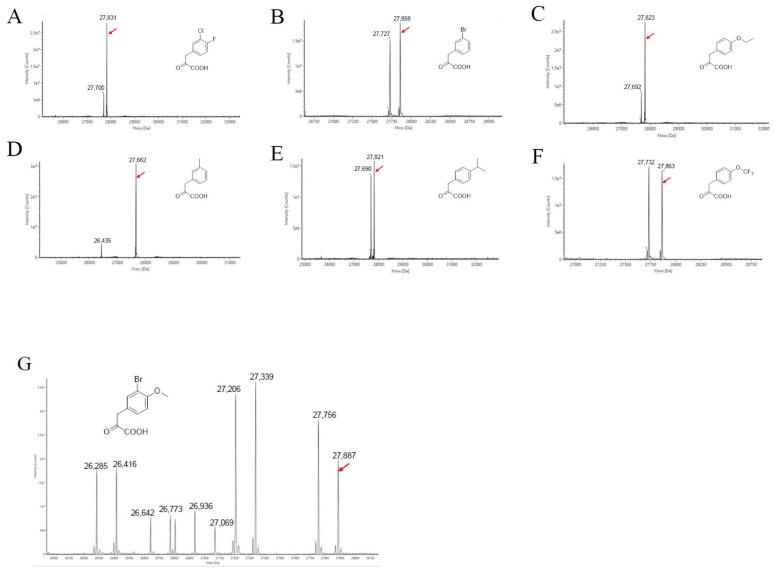
Electrospray ionization mass spectrometry of the eGFPs obtained from *E. coli* DH10B supplemented with the different keto acids. (**A**) eGFP produced with keto acid #2 resulted in a determined MW of 27,831, close to the theoretical MW of 27,831.7. Likewise, #3 (**B**) had a MW of 27,858 (theoretical 27,858.2); #4 (**C**) had a MW of 27,823 (theoretical 27823.3); #5 (**D**) had a MW of 27662, this GFP lacks Met (theoretical 27,793.3); #6 (**E**) had a MW of 27,821 (theoretical 27,821.4); #7 (**F**) had a MW of 27,863 (theoretical 27,863.3); and#8 (**G**) had a MW of 27,887 (theoretical 27,888.2).

The eGFP proteins produced with the different keto acids from #2 to #8 were isolated and purified, and their molecular weights were determined by mass spectrometry (Figure 5). This confirmed the incorporation of the respective ncAAs.

### 3.5. The Evolution for GFPY66H/Y145X

**Figure 6:**
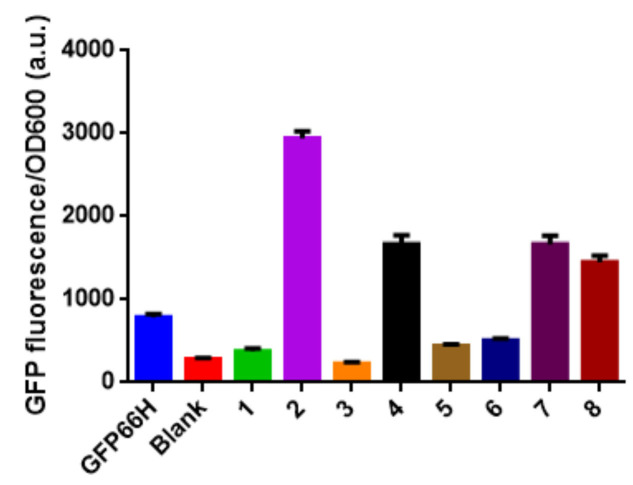
Relative fluorescence intensity of eGFP after incorporation of keto acids #1-#8 combined with Y66H.

It was previously shown that Tyr at position 66 of GFP can be replaced by other aromatic amino acids to form a mature chromophore [33]. When Tyr was replaced by His (Y66H), the protein fluorescence changed from green (510 nm) to blue (450 nm), but the fluorescence intensity was reduced; the interaction with the chromophore changed the state of protonation. In addition, the keto acids were used to introduce their corresponding ncAAs into this background (Figure 6). The effect on the fluorescence of the protein was remarkably different compared to the wildtype protein: when combined with H66Y, #2 resulted in the strongest relative fluorescence, followed by #4, #7, and #8; in contrast, #5 and #6, which had produced high fluorescence in the wildtype form of GFP, now resulted in background levels of fluorescence only, as did #1 and #3. In conclusion, by means of the eight keto acids from #1 to #8, the original Tyr at 145 position of eGFP was replaced by various ncAAs, which changed the fluorescence intensity of the protein. Four of the used keto acids (#2, #4, #7, and #8) resulted in enhanced relative fluorescence value compared with the unmodified protein.

## 4. Discussion

We successfully developed a simple method to genetically encode phenylalanine derivatives that could be incorporated into a protein by supplementation of their α-keto acid precursors. The whole procedure had strong operability and was very suitable for GCE. The green fluorescent protein GFP, which is derived from jellyfish *Aequorea Victoria* and one of the most widely used proteins in biology and medical research, was used as a model system. This protein is commonly used as a marker for gene expression, protein localization and folding, and can be used as a biosensor for non-invasive use [34]. It is a probe for measuring pH, redox potential, metal/halide ion concentration, and protein–protein interaction in vivo [35]. The chromophore is formed by autocatalytic cyclisation of three residues, namely S65, Y66, and G67. The mutation of Tyr at position 145 enhanced the emission of blue fluorescence, which may be due to stable protein folding. Here, we demonstrated that eight different keto acids could be introduced at position 145 of eGFP, resulting in changed fluorescence intensity. In several cases, the introduced keto acid (#2, #4, #7, and #8) resulted in enhanced relative fluorescence values, but the findings depended on the nature of the amino acid at position 66, either Tyr or His.

In the used construct, the synthetase and target protein are under the same arabinose promoter. We aimed for keto acid conversion independent of additional transaminase, for which we identified strains that had sufficient natural transamination activity. This did not apply to all tested strains. For instance, the commercially available strain BL21AI could recognize *p*-MeO-F, but not the corresponding keto acid, indicating that its transamination is relatively weak. This is consistent with the introduction of keto acids in BL21, as described in the literature, that depend on the addition of a transaminase. The *E. coli* strains DH10B and C321.ΔA.expΔP_BAD_ produced sufficient transaminase for our approach. These strains could convert *p*-methoxyphenylpyruvate into *p*-MeO-F and express fluorescent eGFP following incorporation of the nAA into the protein. Without the addition of keto acids or ncAAs, *E. coli* C321.ΔA.expΔP_BAD_ already had a higher background expression than the other tested strains. Since this strain lacks RF-1, the amber codon in the eGFP gene was leaky and did not allow for complete termination of translation, resulting in some background levels. For single-point keto acid introduction, we preferred *E. coli* strain DH10B, while for multi-point keto acid introduction, strain C321.ΔA.expΔP_BAD_ would be a more suitable host.

Amino acids are transported into the cell via a membrane transporter system. The entry of ncAAs into cells can be problematic, as they may not be compatible with existing transport systems. Small molecules, such as keto acids, enter the cell more easily. Keto acids are frequently used for industrial production of amino acids because of easy cell entry, although sometimes detergents are added to pursue a higher conversion rate. In industrial keto acid conversion, the addition of PLP and amino donors is often employed. During their addition in our experimental setup, to our surprise, there was little effect. It is possible that the intracellular concentration of the ncAAs following the conversion of the keto acids was already high to the extent that further optimization could not be achieved.

A total of 13 simple keto acids were synthesized, and these were tested for introduction into eGFP. Nine keto acids were successfully introduced into the protein, and eight resulted in elevated fluorescent levels. This suggests that they were recognized by the membrane transporters, transaminase and MmRS, although the efficiencies of each of these steps were not determined. Most of the incorporated ncAAs contained small-sized hydrophobic side chains, enabling the adopted ability of MmRS. Among them, at the meta position, a halogen (chlorine and bromine) or a small-sized group, such as methyl, was permitted. At the para position, methoxy, ethoxy, isopropyl, and trifluoromethoxy groups were permissive, which can accommodate middle hydrophobic side chains. Derivatives with small substitutions at both the para and meta positions were also incorporated, but large groups or charged groups were not recognized. When incorporated into eGFP, some ncAAs increased the fluorescence of the protein, depending on the nature of the amino acid at position 66.

The amber suppression generally decreases the expression level of ncAA mutants compared to the wildtype; it can be speculated that the expression level of the non-canonical GFP mutants is lower than that of the wildtype or Y66H. Despite this lower expression level, when incorporating keto acids #1, #2, #3, #5, #6, #7, and #8 in eGFP and keto acids #2, #4, #7, and #8 in Y66H, GFP produced higher fluorescence intensities, suggesting that these non-canonical residues improve the fluorescence property of the protein. The lower fluorescence intensity observed with the other keto acids may be due to lower expression levels and/or decreased fluorescence as a result of the mutation. Bromine introduced at the meta position (#3) alone, or in combination with methyl ether at the para position (#8), trifluoromethyl ether, or ethyl ether at the para position (#4), was prioritized.

In conclusion, phenylpyruvate derivatives containing hydrophobic groups or other small groups at the meta and para positions can be recognized by DH10B’s own transaminase and be introduced into eGFP protein using the aminoacyl tRNA system, without the need for additional transaminases. This biosynthetic strategy, based on cheap precursors added to culture media, is expected to significantly reduce the cost and production of recombinant proteins containing ncAA.

## Data Availability

Data is contained within the article, and are available on request from the corresponding author.

## Supplementary Materials

The following are available online at https://www.mdpi.com/article/10.3390/biom11091358/s1, Figures S1–S26: The LC-MS, ^1^H-NMR, ^13^C-NMR data for synthesized compounds, Figures S27–S29: Construction of strain C321.ΔA.expΔPBAD, Figure S30: The SDS-PAGE for eGFP protein purification.
