# Supplementary material for: Convenient Genetic Encoding of Phenylalanine Derivatives through Their α-Keto Acid Precursors
Source: Biomolecules. 2021 Sep 14;11(9):1358. doi: 10.3390/biom11091358 (PMC8470325; doi:10.3390/biom11091358)
Supplement: Supplementary file 1 [file biomolecules-11-01358-s001.zip › biomolecules-1356321-supplementary.pdf]

Supplementary Materials

Convenient genetic encoding of phenylalanine derivatives through their α-keto acid precursors

Li Liu[1], Bohao Wang[1], Sheng Li[1], Fengyuan Xu[1], Qi He1, Chun Pan [2], Xiangdong Gao[1,*], Wenbing Yao[1,*], Xiaoda Song[1,*]

Content:

1.The LC-MS, ^{1}H-NMR, ^{13}C-NMR data for synthesized compounds Page2-19

2. Construction of strain C321.ΔA.expΔPBAD Page 20

3. The SDS-PAGE for eGFP protein purification Page 20

4. The sequence of *Mm*PylRS mutant Page 21

1. The data for synthesized compounds

Mass spectrometry conditions: Dissolve 1.0 mg sample in 1 mL chromatographic methanol to prepare a 1 mg/mL sample solution. The mobile phase gradient is liquid A: 0.1% formic acid aqueous solution, liquid B: acetonitrile. The injection volume is 10μL; the flow rate is 0.8ml/min; the column is Thermo Hypersil GOLD, 250*4.6mm, the particle size is 5μm, and the column temperature is 30°C.

^{13}C-NMR and ^{1}H-NMR spectrum of condensation products: take 10.0 mg of the sample and dissolve it in 550 μL of deuterated DMSO to make a sample solution and send it to the inspection center for analysis.

LC-MS identification of the mobile phase gradient of condensation products.

Time (min)	A liquid (%)	B liquid (%)
0	90	10
8	5	95
10	5	65
10.1	90	10
16	90	10

Compound 1a:

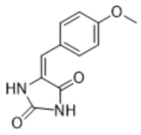

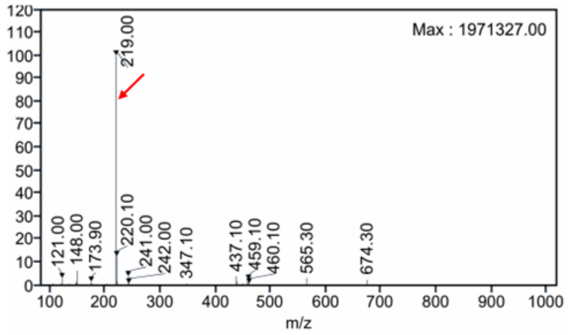

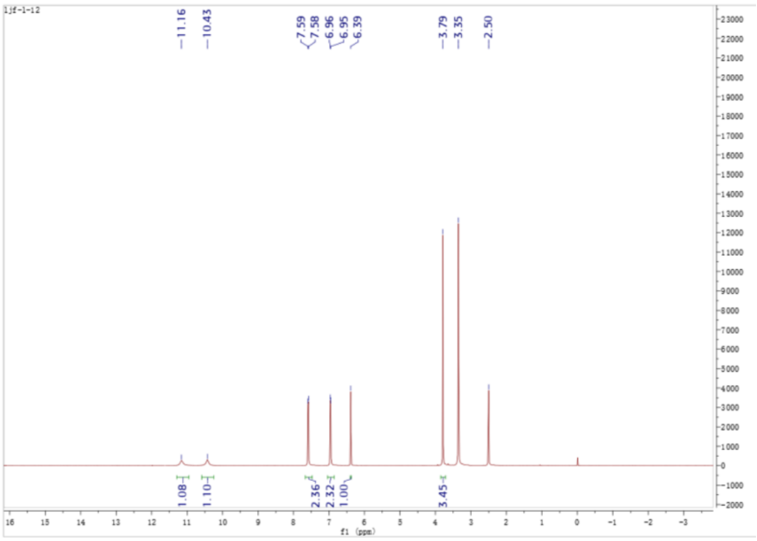

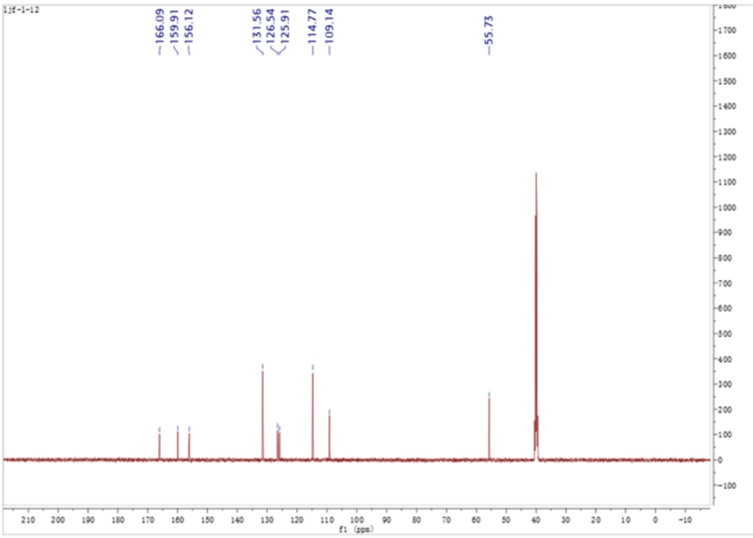

FigureS-1. The LC-MS, ^{1}H-NMR, ^{13}C-NMR data for Compound 1a.

^{1}H-NMR (500 MHz, d6-DMSO) δ 11.16 (s, 1H), 10.43 (s, 1H), 7.58 (d, J = 8.6 Hz, 2H), 6.96 (d, J = 8.5 Hz, 2H), 6.39 (s, 1H), 3.79 (s, 3H). ^{13}C-NMR (126 MHz, d6-DMSO) δ 166.09, 159.91, 156.12, 131.56, 126.54, 125.91, 114.77, 109.14, 55.73. MS (ESI): (m/z) $[M+H]^{+}$ Calcd for C11H11N2O3 219.07; Found 219.00.

Compound 2a：

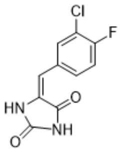

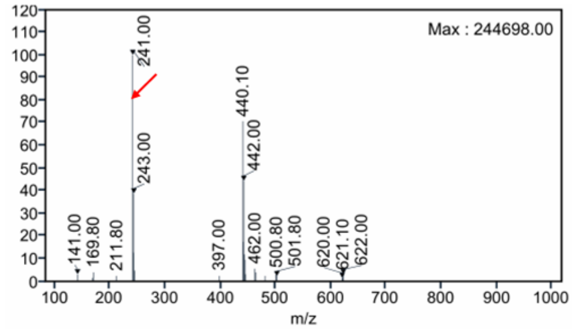

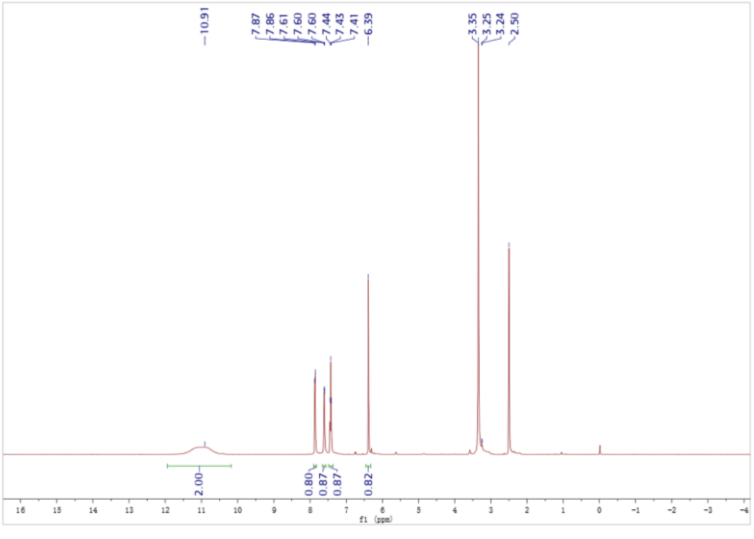

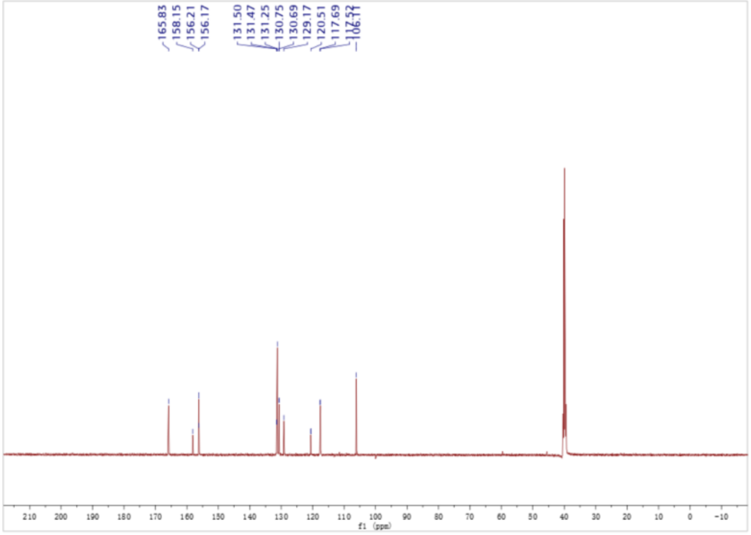

FigureS-2. The LC-MS, ^{1}H-NMR, ^{13}C-NMR data for Compound 2a.

^{1}H-NMR (500 MHz, d6-DMSO) δ 10.91 (s, 2H), 7.86 (d, J= 5.6 Hz, 1H), 7.66 – 7.56 (m, 1H), 7.43 (t, J= 8.9 Hz, 1H), 6.39 (s, 1H).^{13}C-NMR (126 MHz, d6-DMSO) δ 165.84, 157.20 (d, $J_{C8\text{-}F}$ = 243.9 Hz), 156.18, 131.50 (d, $J_{C10\text{-}F}$ = 3.9 Hz), 131.26, 130.73 (d, $J_{C6\text{-}F}$ = 7.5 Hz), 129.19 (d, $J_{C5\text{-}F}$ = 2.2 Hz), 120.59 (d, $J_{C9\text{-}F}$ = 17.7 Hz), 117.62 (d, $J_{C7\text{-}F}$ = 21.2 Hz), 106.13.MS (ESI): (m/z) [M+H]$^{+}$ Calcd for $C_{10}H_7ClFN_2O_2$ 241.02; Found 241.00.

Compound 3a：

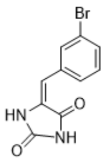

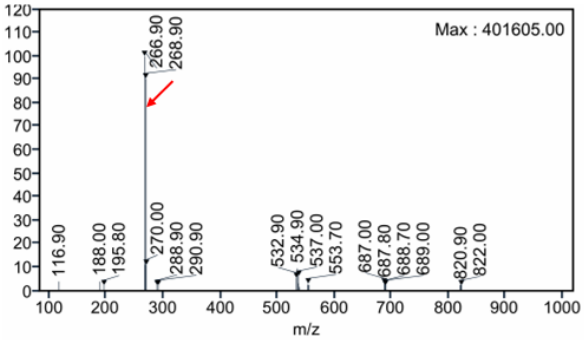

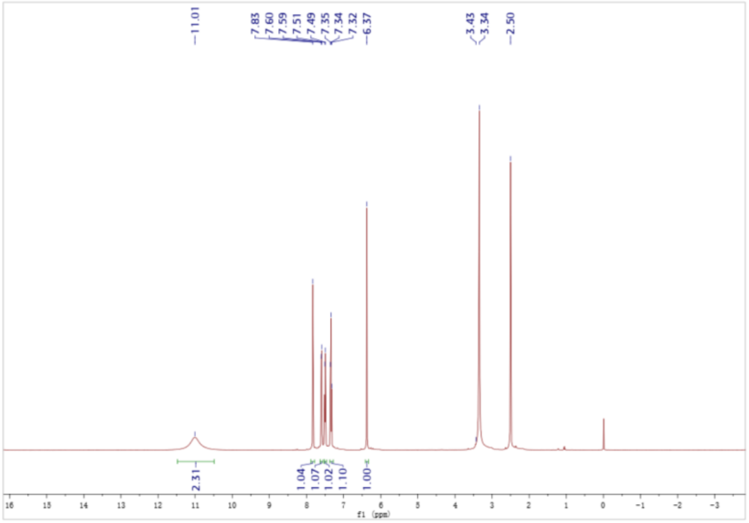

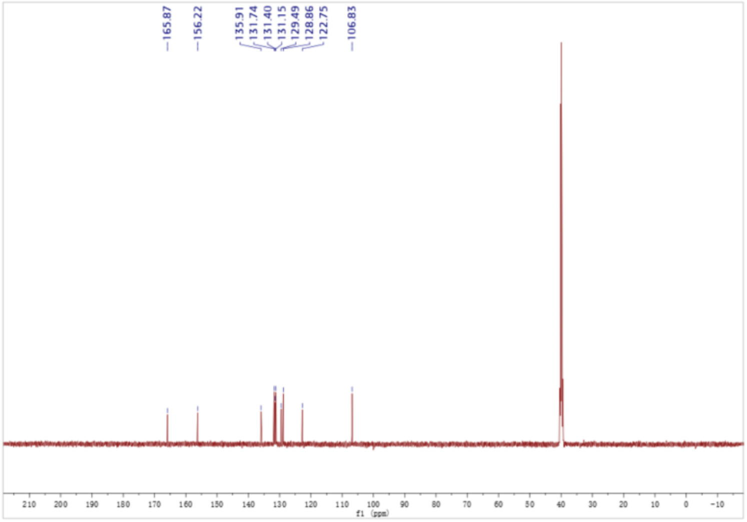

FigureS-3. The LC-MS, ^{1}H-NMR, ^{13}C-NMR data for Compound 3a.

^{1}H-NMR (500 MHz, d6-DMSO) δ 11.01 (s, 2H), 7.83 (s, 1H), 7.59 (d,J= 7.8 Hz, 1H), 7.50 (d,J= 7.9 Hz, 1H), 7.34 (t,J= 7.9 Hz, 1H), 6.37 (s, 1H).^{13}C-NMR (126 MHz, d6-DMSO) δ 165.87, 156.22, 135.91, 131.74, 131.40, 131.15, 129.49, 128.86, 122.75, 106.83.MS (ESI): (m/z) $[M+H]^+$ Calcd for $C_{10}H_8BrN_2O_2$ 266.98; Found 266.90.

Compound 4a：

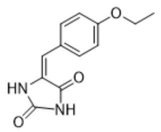

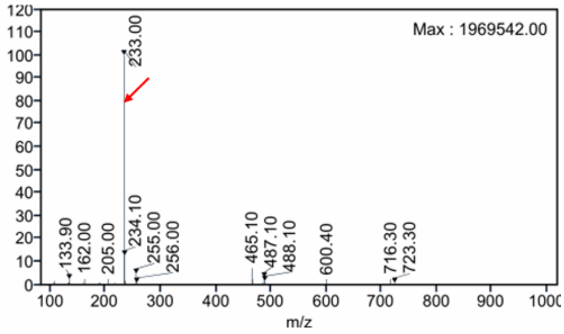

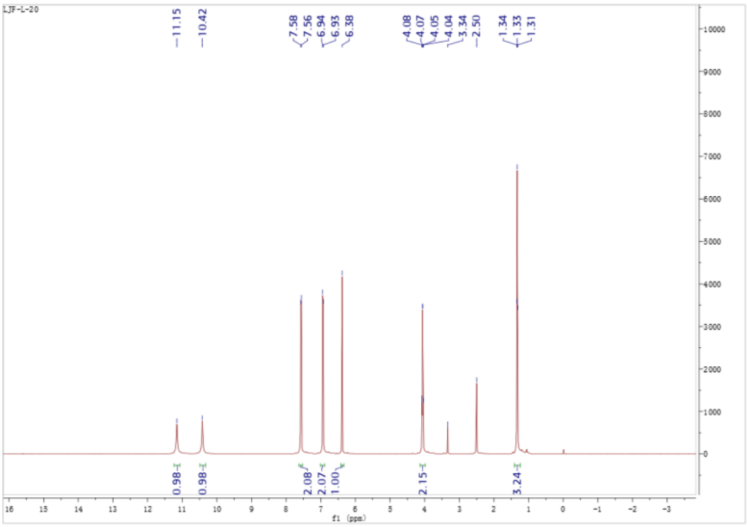

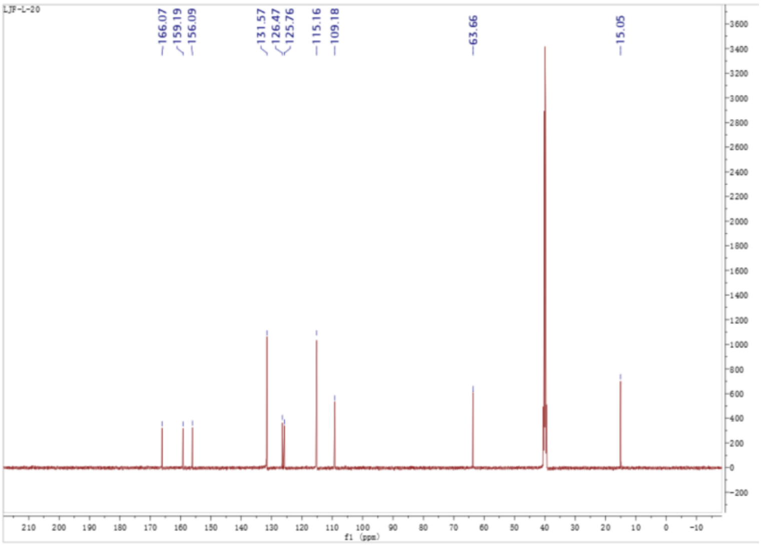

FigureS-4. The LC-MS, ^{1}H-NMR, ^{13}C-NMR data for Compound 4a.

^{1}H-NMR (500 MHz, d6-DMSO) δ 11.15 (s, 1H), 10.42 (s, 1H), 7.57 (d,J= 8.6 Hz, 2H), 6.94 (d,J= 8.6 Hz, 2H), 6.38 (s, 1H), 4.06 (q,J= 6.9 Hz, 2H), 1.33 (t,J= 6.9 Hz, 3H).^{13}C-NMR (126 MHz, d6-DMSO) δ 166.07, 159.19, 156.09, 131.57, 126.47, 125.76, 115.16, 109.18, 63.66, 15.05.MS (ESI): (m/z) $[M+H]^+$ Calcd for $C_{12}H_{13}N_2O_3$ 233.09; Found 233.00.

Compound 5a:

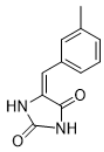

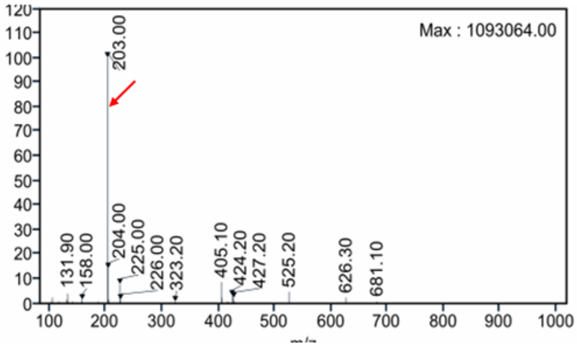

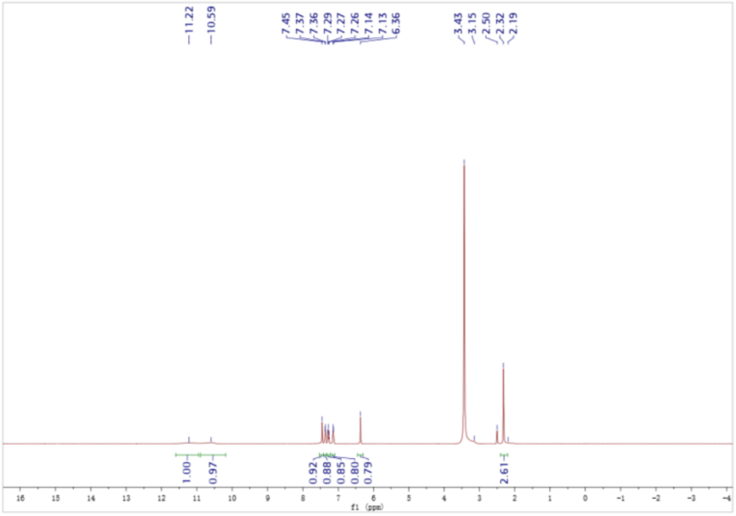

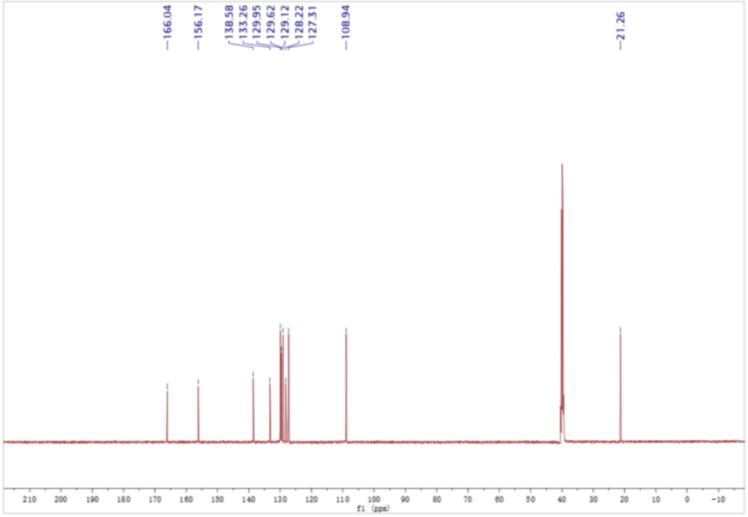

FigureS-5. The LC-MS, ^{1}H-NMR, ^{13}C-NMR data for Compound 5a.

^{1}H-NMR (500 MHz, d6-DMSO) δ 11.22 (s, 1H), 10.59 (s, 1H), 7.45 (s, 1H), 7.37 (d,J= 7.4 Hz, 1H), 7.27 (t,J= 7.5 Hz, 1H), 7.14 (d,J= 7.3 Hz, 1H), 6.36 (s, 1H), 2.32 (s, 3H).^{13}C-NMR (126 MHz, d6-DMSO) δ 166.04, 156.17, 138.58, 133.26, 129.95, 129.62, 129.12, 128.22, 127.31, 108.94, 21.26.MS (ESI): (m/z) $[M+H]^+$ Calcd for $C_{11}H_{11}N_2O_2$ 203.08; Found 203.00.

Compound 6a：

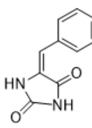

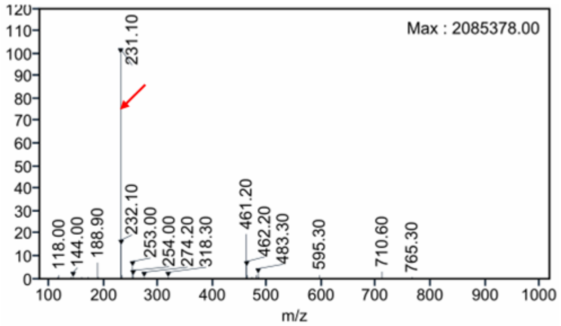

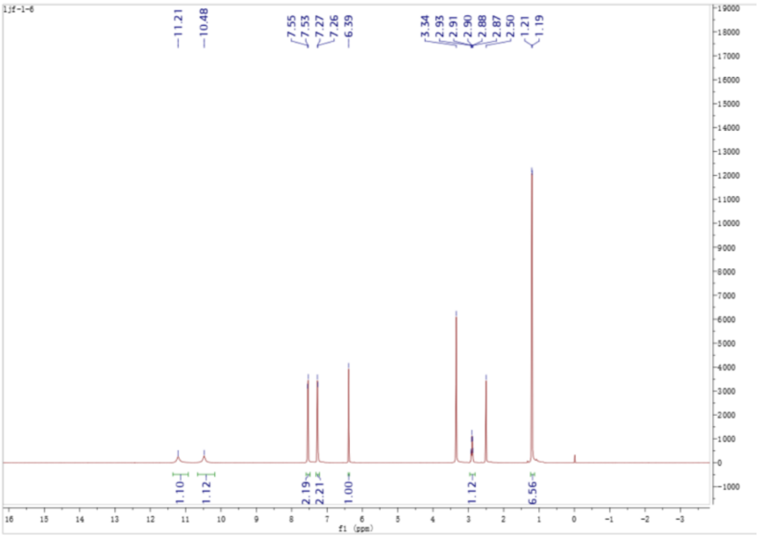

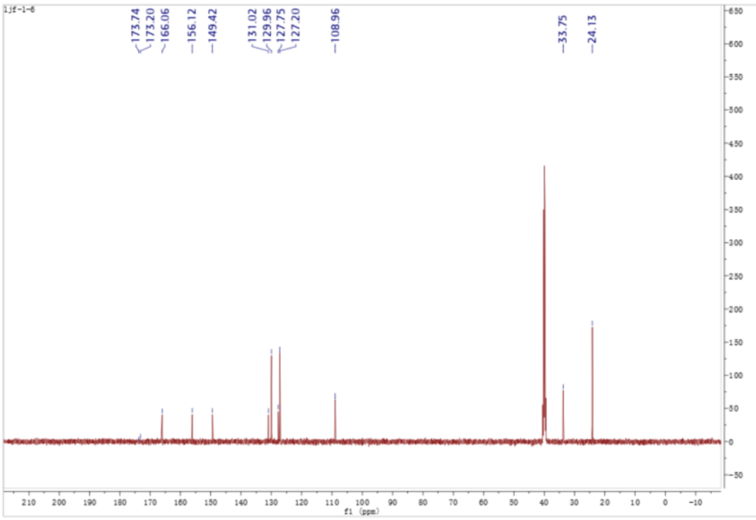

FigureS-6. The LC-MS, ^{1}H-NMR, ^{13}C-NMR data for Compound 6a.

^{1}H-NMR (500 MHz, d6-DMSO) δ 11.21 (s, 1H), 10.48 (s, 1H), 7.54 (d,J= 8.1 Hz, 2H), 7.27 (d,J= 8.1 Hz, 2H), 6.39 (s, 1H), 3.00 – 2.74 (m, 1H), 1.20 (d,J= 6.9 Hz, 6H).^{13}C-NMR (126 MHz, d6-DMSO) δ 173.74, 173.20, 166.06, 156.12, 149.42, 131.02, 129.96, 127.75, 127.20, 108.96, 33.75, 24.13.MS (ESI): (m/z) [M+H]$^+$ Calcd for $C_{13}H_{15}N_2O_2$ 231.12; Found 231.10.

Compound 7a:

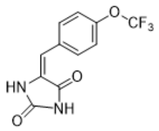

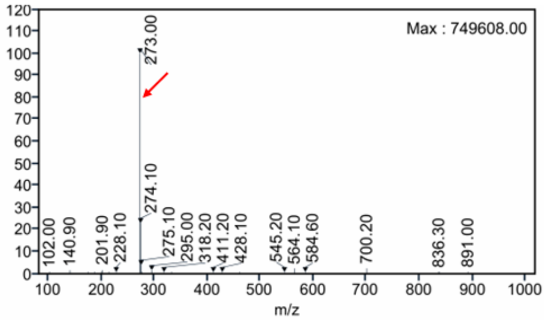

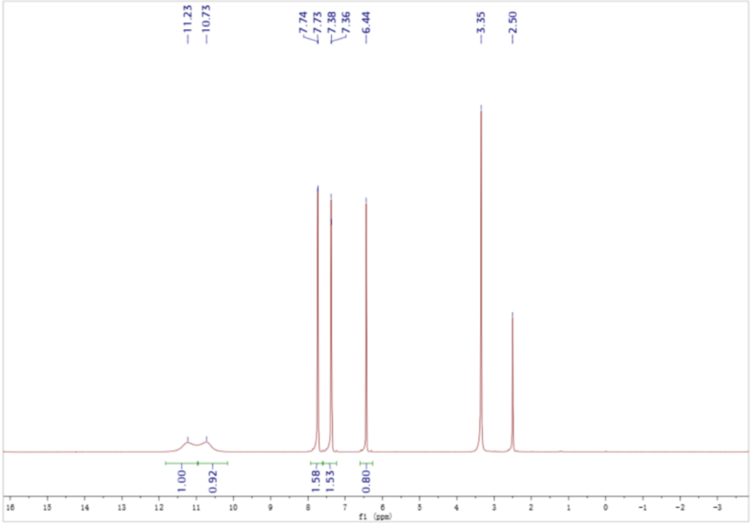

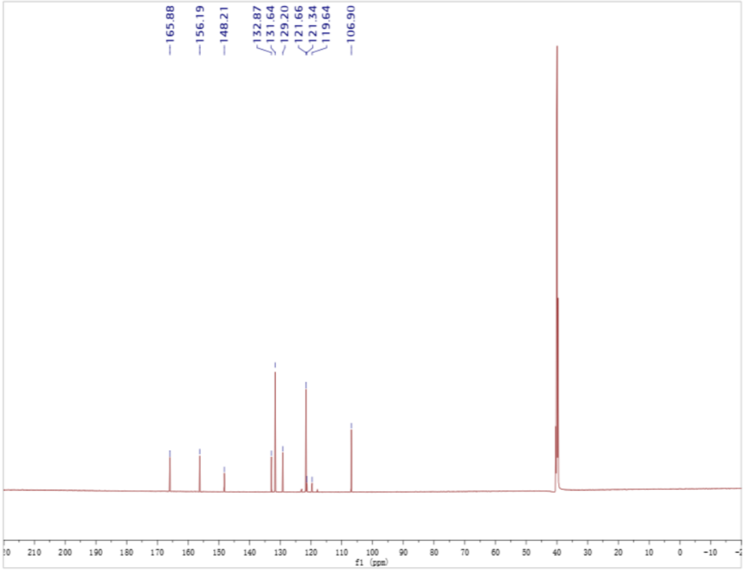

FigureS-7. The LC-MS, ^{1}H-NMR, ^{13}C-NMR data for Compound 7a.

^{1}H-NMR (500 MHz, d6-DMSO) δ 11.23 (s, 1H), 10.73 (s, 1H), 7.73 (d,J= 7.5 Hz, 2H), 7.37 (d,J= 7.6 Hz, 2H), 6.44 (s, 1H).^{13}C-NMR (126 MHz, d6-DMSO) δ 165.88, 156.19, 148.21, 132.87, 131.64, 129.20, 121.66, 121.34, 119.64, 106.90.MS (ESI): (m/z) $[M+H]^+$ Calcd for $C_{11}H_8F_3N_2O_3$ 273.05; Found 273.00.

Compound 8a:

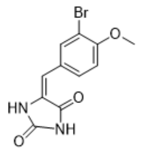

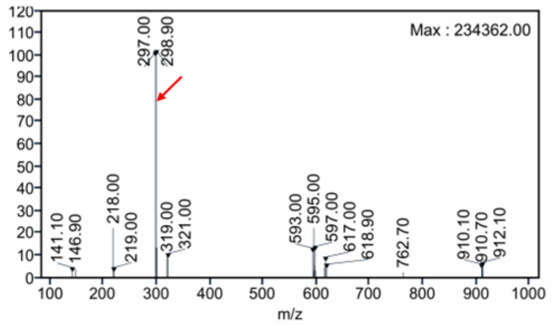

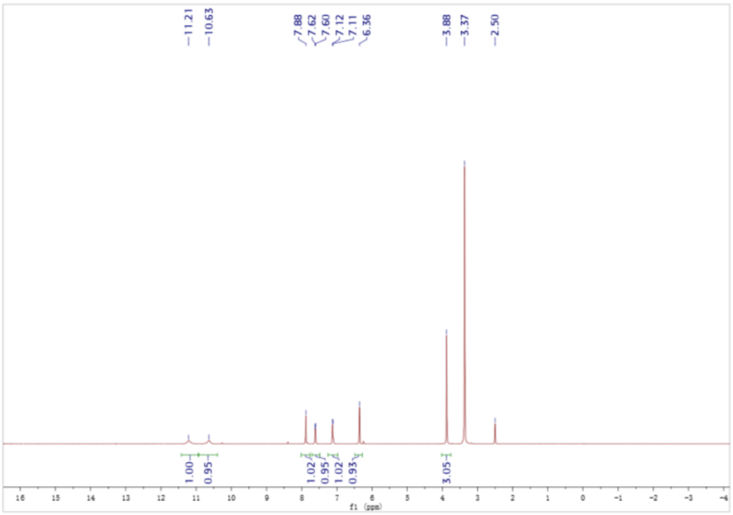

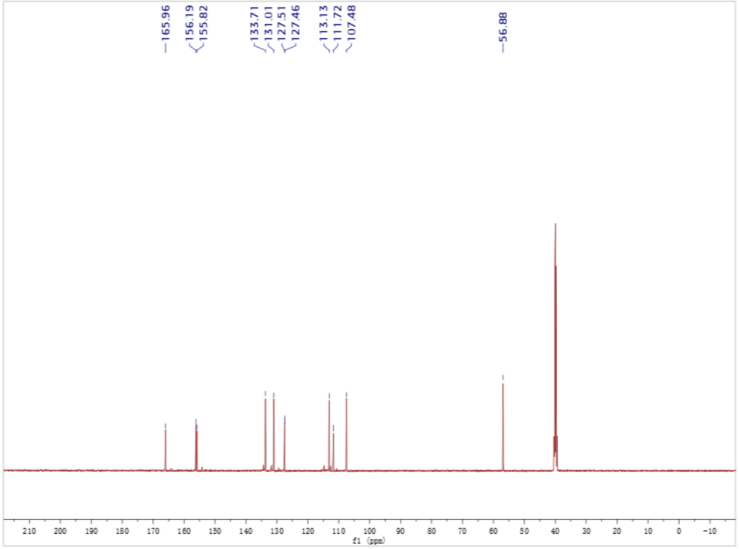

FigureS-8. The LC-MS, ^{1}H-NMR, ^{13}C-NMR data for Compound 8a.

^{1}H-NMR (500 MHz, d6-DMSO) δ 11.21 (s, 1H), 10.63 (s, 1H), 7.88 (s, 1H), 7.61 (d,,J= 8.5 Hz, 1H), 7.11 (d,,J= 8.6 Hz, 1H), 6.36 (s, 1H), 3.88 (s, 3H).^{13}C-NMR (126 MHz, d6-DMSO) δ 165.96, 156.19, 155.82, 133.71, 131.01, 127.51, 127.46, 113.13, 111.72, 107.48, 56.88.MS (ESI): (m/z) $[M+H]^+$ Calcd for $C_{11}H_{10}BrN_2O_3$ 296.99; Found 297.00.

Compound 9a:

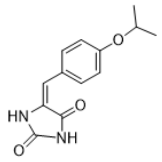

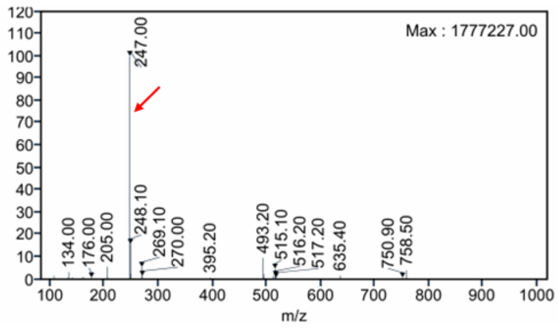

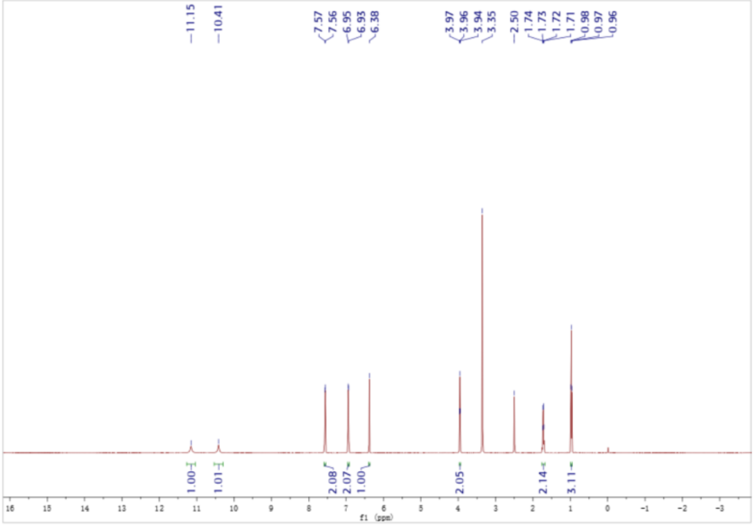

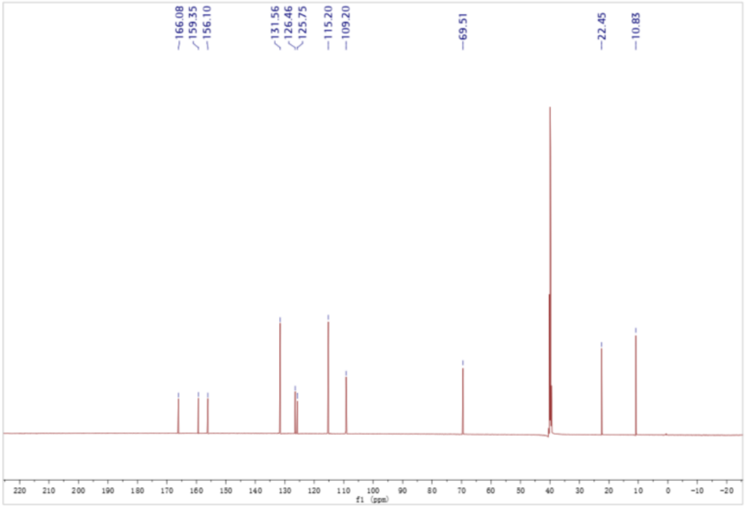

FigureS-9. The LC-MS, ^{1}H-NMR, ^{13}C-NMR data for Compound 9a.

^{1}H-NMR (500 MHz, d6-DMSO) δ 11.15 (s, 1H), 10.41 (s, 1H), 7.57 (d,*J*= 8.7 Hz, 2H), 6.94 (d,*J*= 8.7 Hz, 2H), 6.38 (s, 1H), 3.96 (t,*J*= 6.5 Hz, 2H), 1.72 (dd,*J*= 14.0, 6.9 Hz, 2H), 0.97 (t,*J*= 7.4 Hz, 3H).^{13}C-NMR (126 MHz, d6-DMSO) δ 166.08, 159.35, 156.10, 131.56, 126.46, 125.75, 115.20, 109.20, 69.51, 22.45, 10.83.MS (ESI): (m/z) $[M+H]^+$ Calcd for $C_{13}H_{15}N_2O_3$ 247.11; Found 247.00.

Compound 10a:

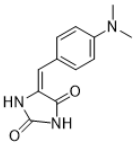

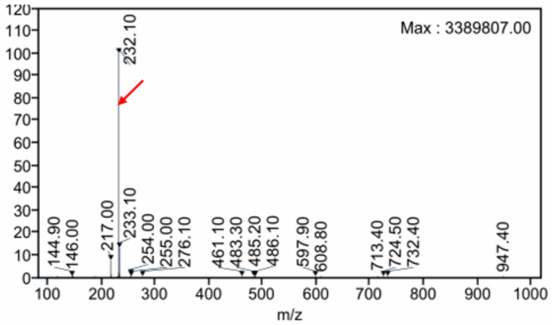

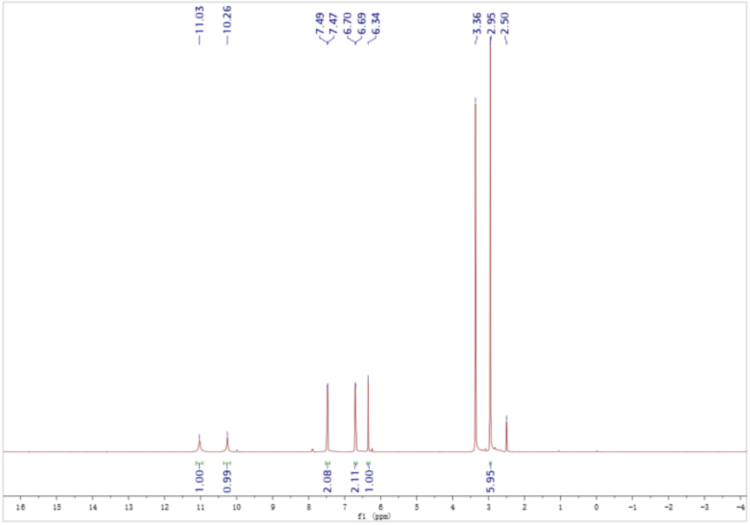

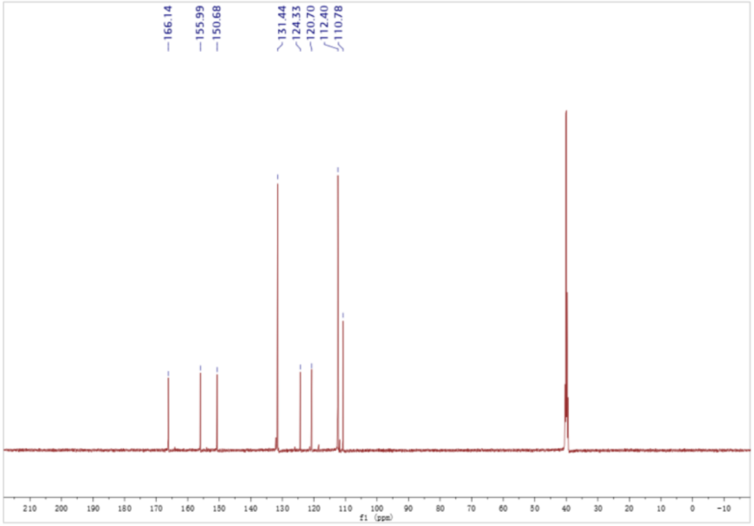

FigureS-10. The LC-MS, ^{1}H-NMR, ^{13}C-NMR data for Compound 10a.

^{1}H-NMR (500 MHz, d6-DMSO) δ 11.03 (s, 1H), 10.26 (s, 1H), 7.48 (d,J= 8.4 Hz, 2H), 6.70 (d,J= 8.4 Hz, 2H), 6.34 (s, 1H), 2.95 (s, 6H).^{13}C-NMR (126 MHz, d6-DMSO) δ 166.14, 155.99, 150.68, 131.44, 124.33, 120.70, 112.40, 110.78.MS (ESI): (m/z) $[M+H]^{+}$ Calcd for $C_{12}H_{14}N_{3}O_{2}$ 232.11; Found 232.10.

Compound 11a:

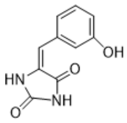

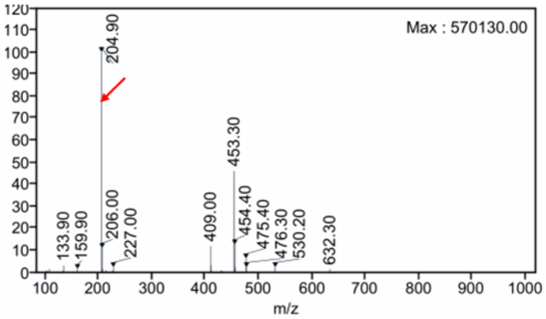

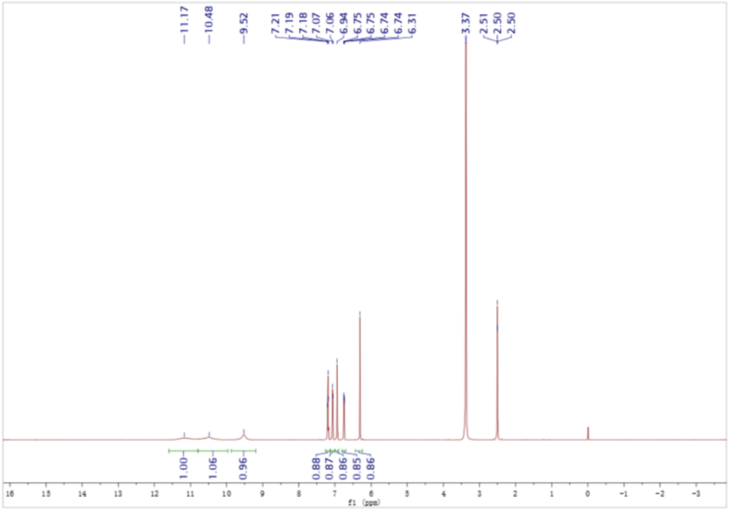

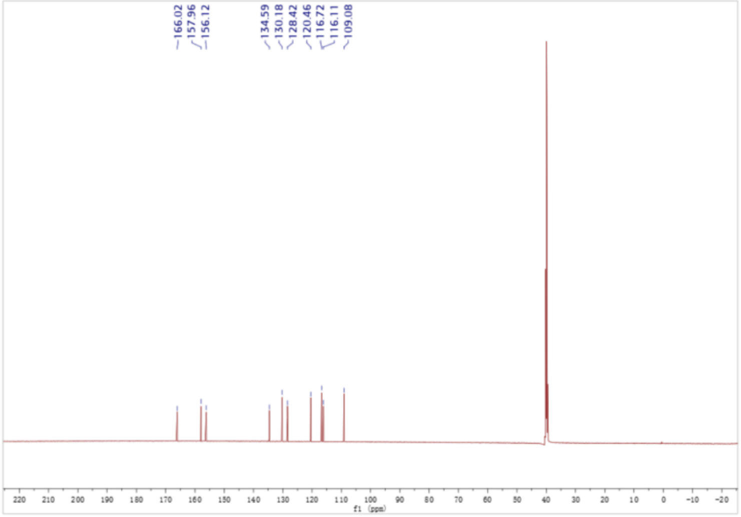

FigureS-11. The LC-MS, ^{1}H-NMR, ^{13}C-NMR data for Compound 11a.

^{1}H-NMR (500 MHz, d6-DMSO) δ 11.17 (s, 1H), 10.48 (s, 1H), 9.52 (s, 1H), 7.19 (t, J= 7.9 Hz, 1H), 7.06 (d, J= 7.7 Hz, 1H), 6.94 (s, 1H), 6.81 – 6.70 (m, 1H), 6.31 (s, 1H).^{13}C-NMR (126 MHz, d6-DMSO) δ 166.02, 157.96, 156.12, 134.59, 130.18, 128.42, 120.46, 116.72, 116.11, 109.08.MS (ESI): (m/z) $[M+H]^{+}$ Calcd for $C_{10}H_{9}N_{2}O_{3}$ 205.06; Found 204.90

Compound 12a：

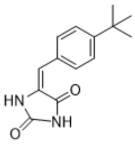

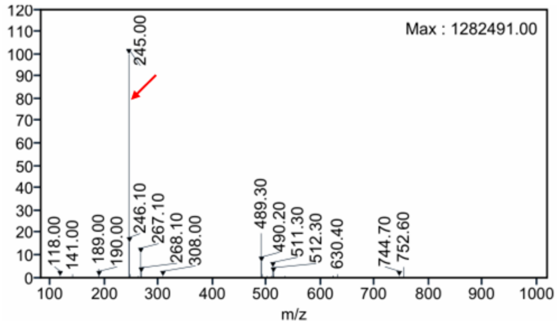

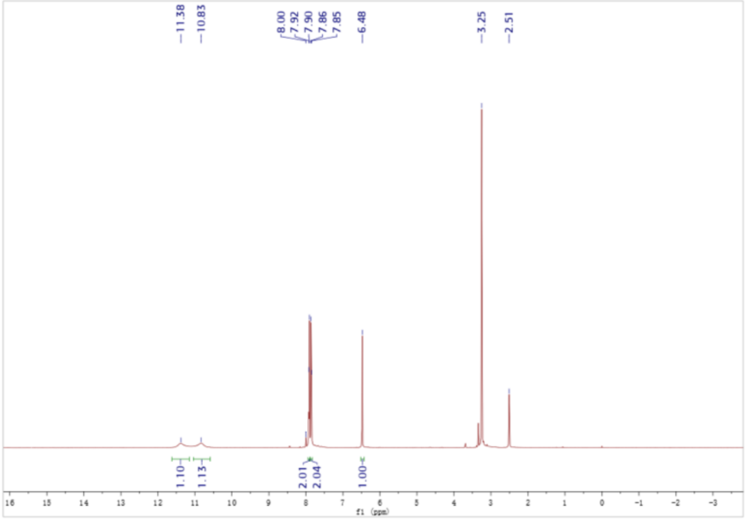

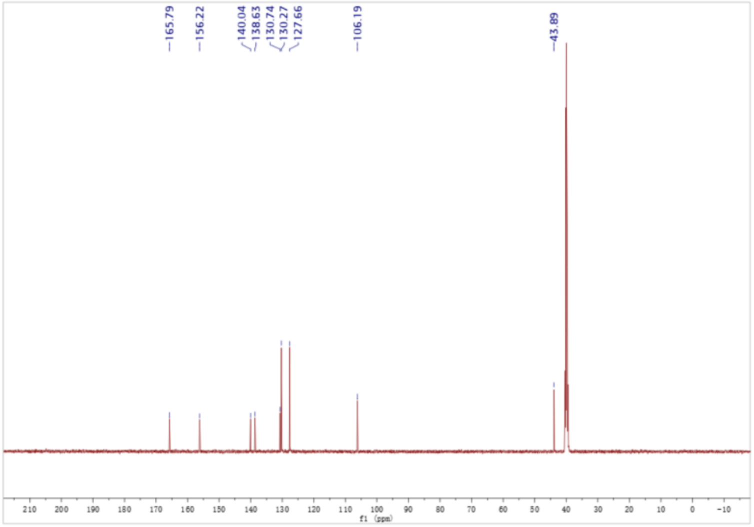

FigureS-12. The LC-MS, ^{1}H-NMR, ^{13}C-NMR data for Compound 12a.

^{1}H-NMR (500 MHz, d6-DMSO) δ 11.38 (s, 1H), 10.83 (s, 1H), 7.91 (d,J= 8.3 Hz, 2H), 7.86 (d,J= 8.3 Hz, 2H), 6.48 (s, 1H).^{13}C-NMR (126 MHz, d6-DMSO) δ 165.79, 156.22, 140.04, 138.63, 130.74, 130.27, 127.66, 106.19, 43.89.MS (ESI): (m/z) $[M+H]^{+}$ Calcd for $C_{14}H_{17}N_{2}O_{2}$ 244.13; Found 245.00.

Compound 13a:

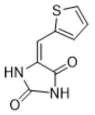

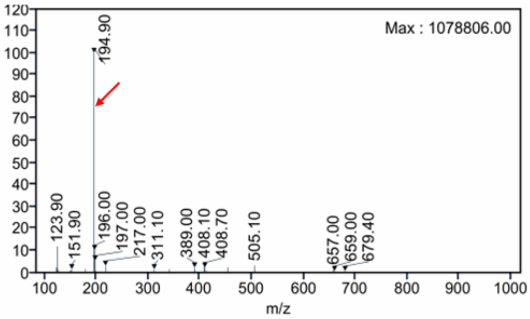

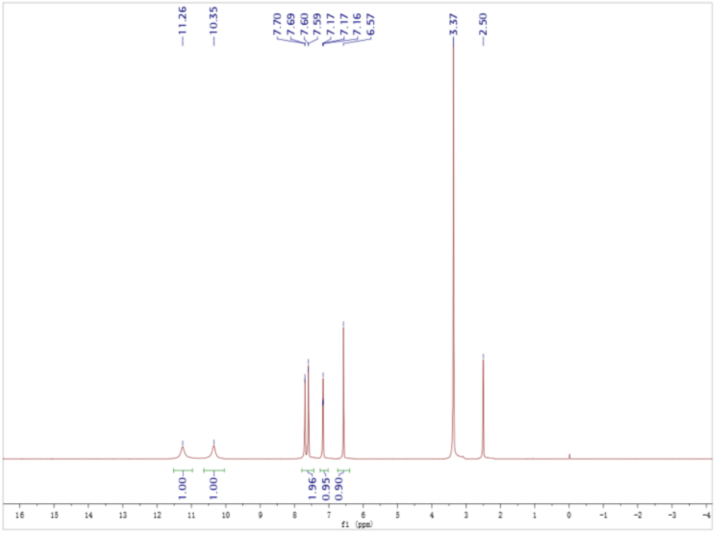

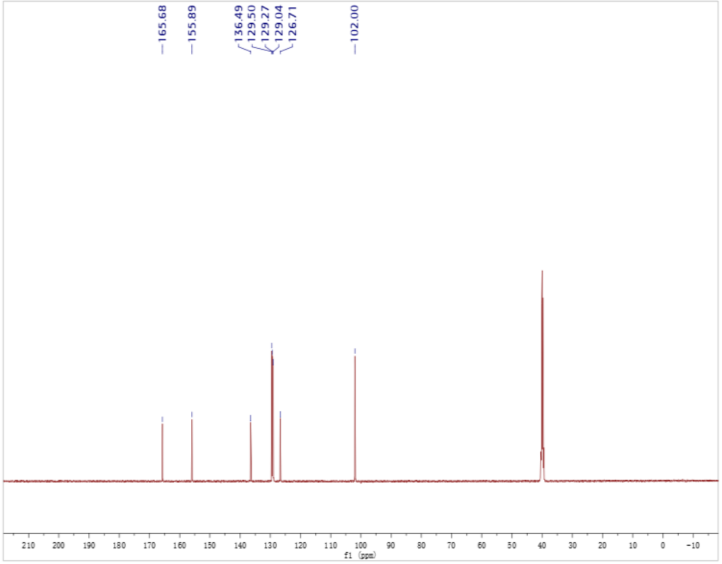

FigureS-13. The LC-MS, ^{1}H-NMR, ^{13}C-NMR data for Compound 13a.

^{1}H-NMR (500 MHz, d6-DMSO) δ 11.26 (s, 1H), 10.35 (s, 1H), 7.64 (dd,,*J*= 49.4, 3.8 Hz, 2H), 7.17 (t,,*J*= 3.8 Hz, 1H), 6.57 (s, 1H).^{13}C-NMR (126 MHz, d6-DMSO) δ 165.68, 155.89, 136.49, 129.50, 129.27, 129.04, 126.71, 102.00.MS (ESI): (m/z) $[M+H]^+$ Calcd for $C_8H_7N_2O_2S$ 195.02; Found 194.90

Compound 1b:

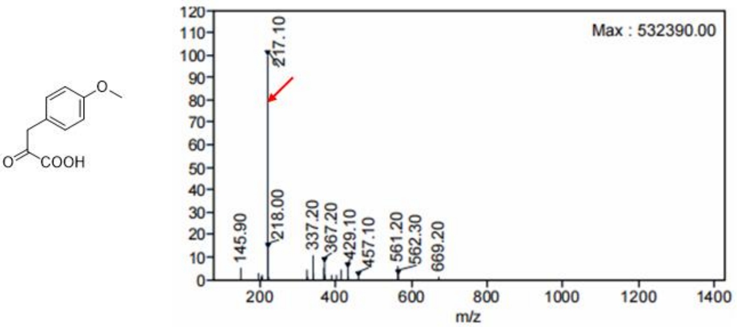

FigureS-14. The LC-MS data for Compound 1b.

MS (ESI): (m/z) $[M+Na]^+$ Calcd for $C_{10}H_9NaO_4$ 216.04; Found 217.10.

Compound 2b:

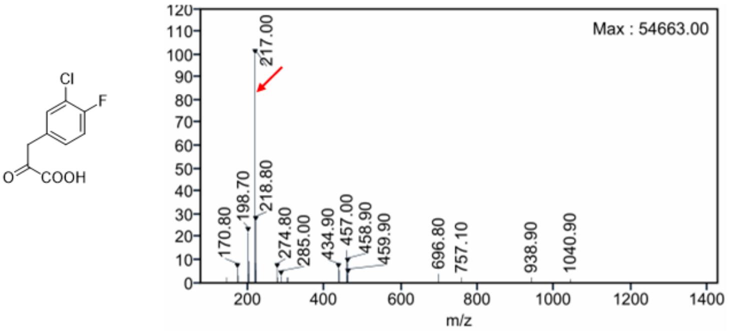

FigureS-15. The LC-MS data for Compound 2b.

MS (ESI): (m/z) $[M+H]^+$ Calcd for $C_9H_6ClFO_3$ 216.00; Found 217.00.

Compound 3b:

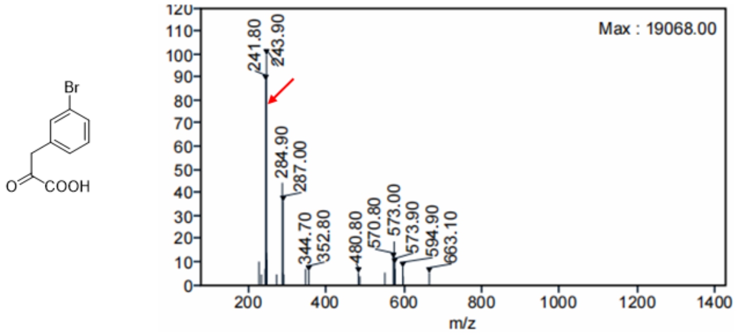

FigureS-16. The LC-MS data for Compound 3b.

MS (ESI): (m/z) $[M+H]^+$ Calcd for $C_9H_7BrO_3$ 241.96; Found 243.90.

Compound 4b:

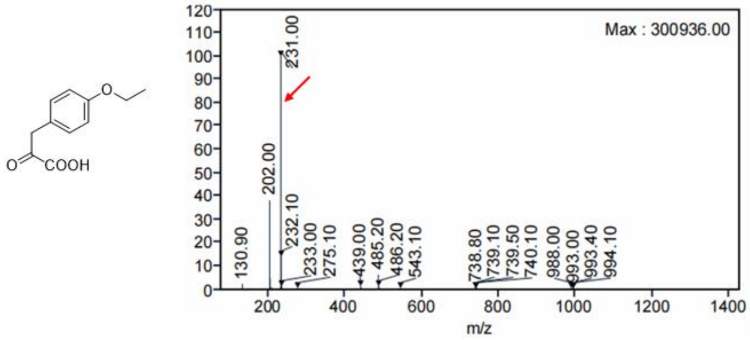

FigureS-17. The LC-MS data for Compound 4b.

MS (ESI): (m/z) $[M+Na]^+$ Calcd for $C_{11}H_{11}NaO_4$ 230.06; Found 231.00.

Compound 5b:

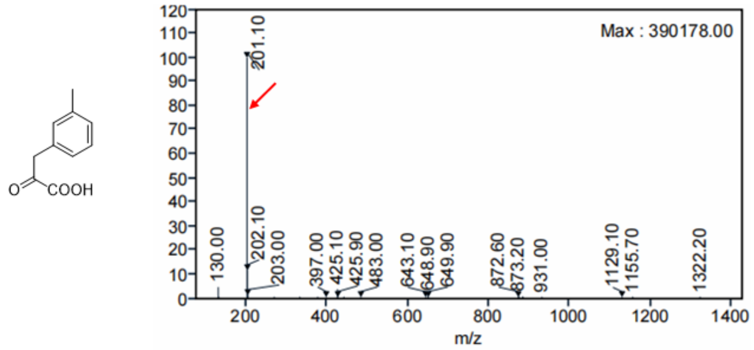

FigureS-18. The LC-MS data for Compound 5b.

MS (ESI): (m/z) $[M+Na]^+$ Calcd for $C_{10}H_9NaO_3$ 200.04; Found 201.10.

Compound 6b:

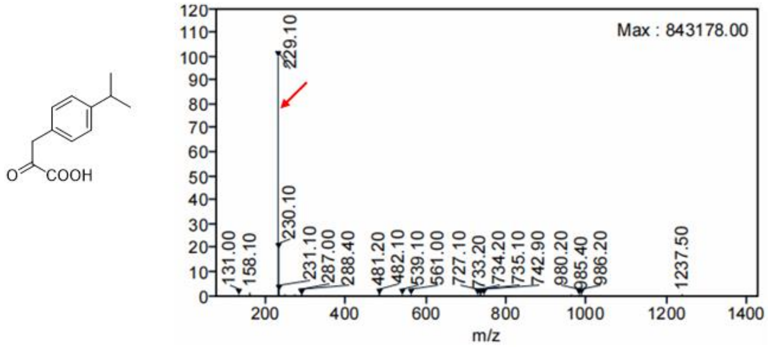

FigureS-19. The LC-MS data for Compound 6b.

MS (ESI): (m/z) $[M+Na]^+$ Calcd for $C_{12}H_{13}NaO_3$ 228.09; Found 229.10.

Compound 7b:

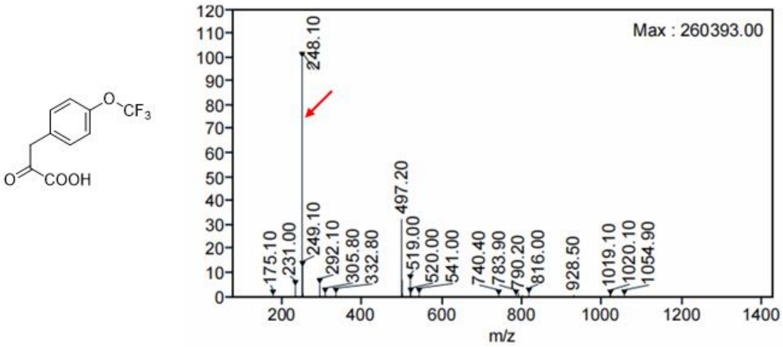

FigureS-20. The LC-MS data for Compound 7b.

MS (ESI): (m/z) $[M]^+$ Calcd for $C_{10}H_7F_3O_4$ 248.03; Found 248.10.

Compound 8b:

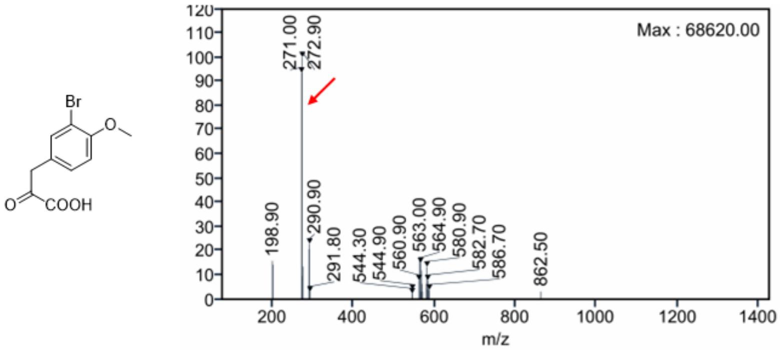

FigureS-21. The LC-MS data for Compound 8b.

MS (ESI): (m/z) $[M+H]^+$ Calcd for $C_{10}H_9BrO_4$ 271.97; Found 272.90.

Compound 9b:

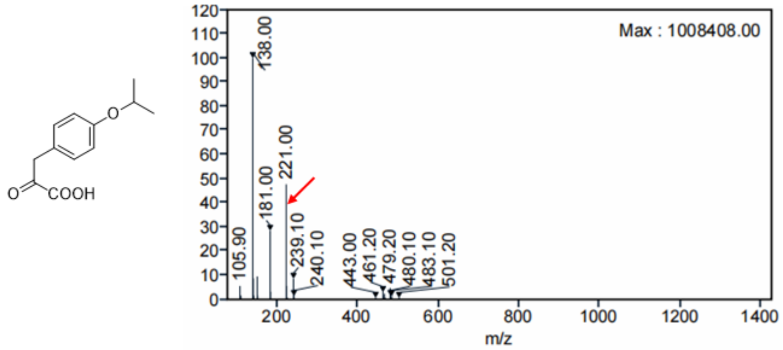

FigureS-22. The LC-MS data for Compound 9b.

MS (ESI): (m/z) $[M-H]^-$ Calcd for $C_{12}H_{14}O_4$ 222.09; Found 221.00.

Compound 10b:

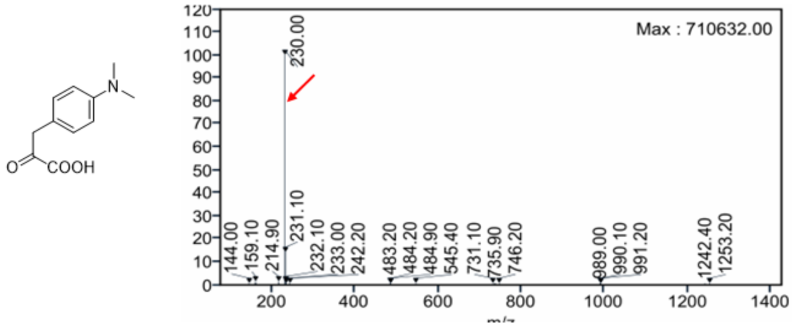

FigureS-23. The LC-MS data for Compound 10b.

MS (ESI): (m/z) $[M+Na]^+$ Calcd for $C_{11}H_{12}NNaO_3$ 229.07; Found 230.00.

Compound 11b:

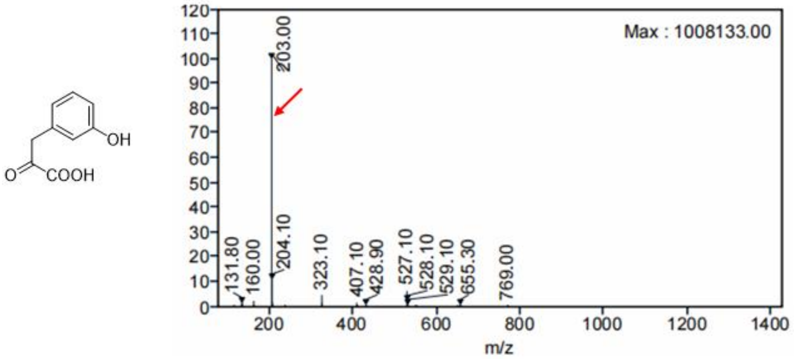

FigureS-24. The LC-MS data for Compound 11b.

MS (ESI): (m/z) $[M+Na]^+$ Calcd for $C_9H_7NaO_4$ 202.02; Found 203.00.

Compound 12b:

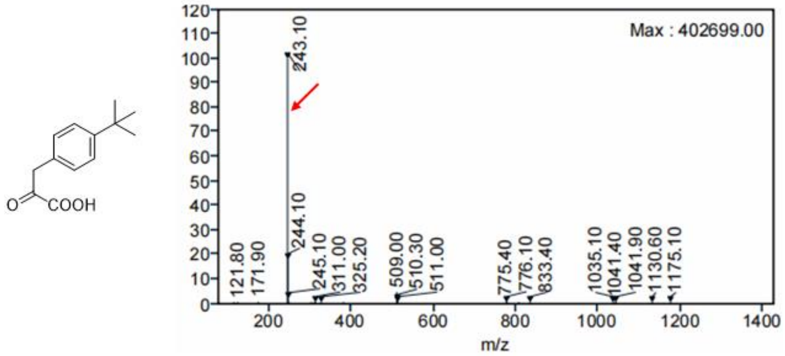

FigureS-25. The LC-MS data for Compound 12b.

MS (ESI): (m/z) $[M+Na]^+$ Calcd for $C_{13}H_{15}NaO_3$ 242.09; Found 243.00.

Compound 13b:

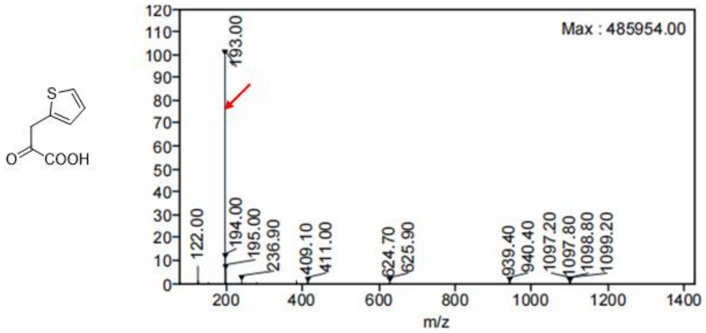

FigureS-26. The LC-MS data for Compound 13b.

MS (ESI): (m/z) $[M+Na]^+$ Calcd for $C_7H_5NaO_3S$ 191.99; Found 193.00.

2. Construction of strain C321.ΔA.expΔPBAD

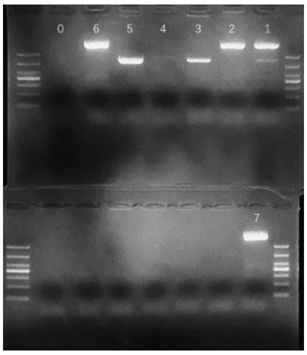

Figure S-27. The strain PCR result for knockout. Lane 0 was negative control; Lane 1, 4 was mixed bacteria; Lane 3 and 5 were knockout failure strains; Lane2, 6 and 7 were succeed strains.

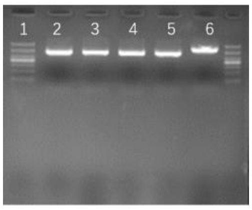

Figure S-28. The strain PCR result for antibiotic removed. Lane 2-5 was the antibiotic removed succeed Lane 6 was the negative control.

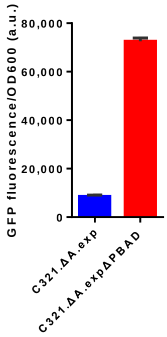

Figure S-29. The bacterial carry pBAD-eGFP plasmid with arabinose induced. 1. Strain C321.ΔA.exp 2. Strain C321.ΔA.expΔPBAD

3. The SDS-PAGE for eGFP protein purification

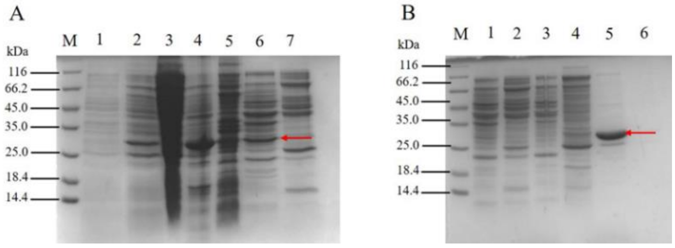

Figure S-30. A. SDS-PAGE for Q column purification. M. Standard molecular weight protein Marker 26610; Lane1. Whole bacteria before EGFP induction; Lane2. Whole bacteria after EGFP induction; Lane3. EGFP high-pressure breaking precipitation; Lane4. EGFP high-pressure breaking supernatant; Lane5 EGFP 20mM Tris.HCl /50mM NaCl elution; Lane6. EGFP 20mM Tris.HCl /100mM NaCl elution; Lane7. EGFP 20mM Tris.HCl /300mM NaCl

elution; B. SDS-PAGE for Co column purification. M. Standard molecular weight protein Marker 26610; Lane1-3. EGFP sample flow through; Lane4. EGFP 20mM Tris.HCl/500mM NaCl/15mM imidazole elution; Lane5. EGFP 20mM Tris.HCl/500mM NaCl/100mM imidazole elution; Lane5-7. EGFP 20mM Tris.HCl /500mM NaCl/300mM imidazole elution

4. The sequence of *Mm*PylRS

MDKKPLNTLISATGLWMSRTGTIHKIKHHEVSRSKIYIEMACGDHLVVNNSRSSRTARALR
HHKYRKTCKRCRVSDEDLNKFLTKANEDQTSVKVKVVSAPTRTKKAMPKSVARAPKPLE
NTEAAQAQPSGSKFSPAIPVSTQESVSVPASVSTSISSISTGATASALVKGNTNPITSMSAPV
QASAPALTKSQTDRLEVLLNPKDEISLNSGKPFRELESELLSRRKKDLQQIYAEERENYLGK
LEREITRFFVDRGFLEIKSPILIPLEYIERMGIDNDTELSKQIFRVDKNFCLRPMLAPNLYNYL
RKLDRALPDPIKIFEIGPCYRKESDGKEHLEEFTMLAFAQMGSGCTRENLESIITDFLNHLGI
DFKIVGDSCMVYGDTLDVMHGDLELSSAVVGPIPLDREWGIDKPWIGAGFGLERLLKVK
HDFKNIKRAARSESYYNGISTNL
